# Evolution of a complex phenotype with biphasic ontogeny: Contribution of development versus function and climatic variation to skull modularity in toads

**DOI:** 10.1002/ece3.3592

**Published:** 2017-11-07

**Authors:** Monique Nouailhetas Simon, Gabriel Marroig

**Affiliations:** ^1^ Departamento de Genética e Biologia Evolutiva Instituto de Biociências Universidade de São Paulo São Paulo Brasil

**Keywords:** adaptive landscape, anuran metamorphosis, morphological integration, stabilizing selection

## Abstract

The theory of morphological integration and modularity predicts that if functional correlations among traits are relevant to mean population fitness, the genetic basis of development will be molded by stabilizing selection to match functional patterns. Yet, how much functional interactions actually shape the fitness landscape is still an open question. We used the anuran skull as a model of a complex phenotype for which we can separate developmental and functional modularity. We hypothesized that functional modularity associated to functional demands of the adult skull would overcome developmental modularity associated to bone origin at the larval phase because metamorphosis would erase the developmental signal. We tested this hypothesis in toad species of the *Rhinella granulosa* complex using species phenotypic correlation pattern (P‐matrices). Given that the toad species are distributed in very distinct habitats and the skull has important functions related to climatic conditions, we also hypothesized that differences in skull trait covariance pattern are associated to differences in climatic variables among species. Functional and hormonal‐regulated modules are more conspicuous than developmental modules only when size variation is retained on species P‐matrices. Without size variation, there is a clear modularity signal of developmental units, but most species have the functional model as the best supported by empirical data without allometric size variation. Closely related toad species have more similar climatic niches and P‐matrices than distantly related species, suggesting phylogenetic niche conservatism. We infer that the modularity signal due to embryonic origin of bones, which happens early in ontogeny, is blurred by the process of growth that occurs later in ontogeny. We suggest that the species differing in the preferred modularity model have different demands on the orbital functional unit and that species contrasting in climate are subjected to divergent patterns of natural selection associated to neurocranial allometry and T3 hormone regulation.

## 1 INTRODUCTION

A central idea in multivariate evolution is that changes in one trait may not be independent of changes in other traits within a complex structure, a phenomenon known as phenotypic integration (Armbruster et al., 2014; Berg, 1960; Olson & Miller, 1958; Pigliucci & Preston, 2004). The related concept of modularity refers to the relative independence among groups of traits due to the distribution of allelic effects, in which pleiotropic effects (alleles affecting more than one trait) are more numerous or stronger within a unit when compared to between units (Klingenberg, 2008; Waddington, 1957; Wagner, 1996; Wagner, Pavlicev, & Cheverud, 2007). Modular phenotypes have been documented in distinct levels of biological systems, from gene expression and molecules to morphology (see review by Wagner, Pavlicev & Cheverud, 2007). The pattern and magnitude of integration among traits may have important ecological and evolutionary consequences, including the coordinate change of biological units (Cheverud, 1984) and a higher adaptability of the phenotypes because changes of one module could occur with limited interference in other modules composing the same system (Wagner & Altenberg, 1996; West‐Eberhard, 2003).

In order to understand the evolution of complex phenotypes, one must uncover the processes that structure the interactions among traits at the population level. Olson & Miller (1958) proposed that traits sharing a developmental pathway and/or exerting a common function should be more integrated among themselves than with traits from distinct developmental origins or acting on distinct functions. Riedl (1977, 1978), followed by Cheverud (1982, 1984), suggested that the genetic basis of development would evolve to match the shape of the fitness landscape, in which traits interacting to perform the same function would become controlled by the same set of pleiotropic genes (Lande, 1980). That is, stabilizing selection imposed by the epigenetic developmental system would favor coadaptation of traits belonging to the same unit to warrant proper functioning of organisms (Cheverud, 1984; Hansen & Houle, 2004; Riedl, 1978). The fitness surface molding phenotypic correlations is composed of both internal and external stabilizing selection (Cheverud, 1984). The internal selection acts against changes that impair proper functioning of one trait in regard to another due to negative fitness consequences for the organism and is independent of the external environment (Schwenk & Wagner, 2001); whereas the external selection depends on the interaction between phenotype and environment (Cheverud, 1984). Yet, whether functional interactions among traits actually influence the fitness landscape is still unknown (Klingenberg, Debat, & Roff, 2010; Young & Badyaev, 2006; Zelditch & Swiderski, 2011), partially because organisms have been already potentially shaped by this interplay of development and function. When population mean fitness depends on certain traits being correlated with other traits in the same functional module, we expect such a fitness landscape to shape genetic correlations so that they mimic the functional relations among traits (Cheverud, 1982, 1984).

In this work, we used the anuran amphibian skull as a model of a complex phenotype that provides the opportunity to test the support for developmental versus functional processes in shaping trait correlation patterns. Piekarski, Gross, & Hanken (2014) published a cranial neural crest (CNC) fate map study that revealed a remarkable difference in the contribution of the CNC to the bony skull between the anuran *Xenopus laevis* and the rest of the tetrapods. Virtually, all skull bones are derived from CNC streams in *X. laevis*, whereas in the other tetrapods, there is a clear separation between bones derived from the CNC (face) and from the paraxial mesoderm (neurocranium). This difference in skull development in anurans allows for the construction of competing modularity models, as the functional hypothetical modules do not completely coincide with the developmental modules. The use of the anuran skull also adds another interesting aspect to the study of modularity: the occurrence of a biphasic ontogeny mediated by metamorphosis, which is an extreme developmental remodeling process, promoting deep changes in cranial morphology (Hanken & Summers, 1988; Rose & Reiss, 1993. In view of the Palimpsest model, where distinct developmental processes overlap in space and time throughout ontogeny producing divergent covariation patterns that overwrite each other in the adult skull (Hallgrímsson et al., 2009), we may interpret metamorphosis as a rupture in the timing of development. Metamorphosis mediates the transition of the larval aquatic phase to an adult terrestrial phase, where the chondrocranium that performed tadpole functions undergoes several morphological changes to become the bony skull that performs adult functions (Hanken & Summers, 1988; Kerney et al., 2012). We thus hypothesized that the functional demands of the newly formed adult skull would impose a correlation pattern among skull traits that overrides the modularity signal due to the earlier developmental processes associated to the larval phase. Therefore, we expect skull trait correlations in the adult anurans to reflect functional modularity more than developmental modularity related with embryonic origins of the bones.

We tested the relevance of development versus function structuring skull trait correlations in a comparative framework by working with the anuran toad species of the *Rhinella granulosa* complex (Gallardo 1965, Narvaes & Rodrigues, 2009). These toad species are distributed in habitats with distinct climatic regimes, and we previously showed that part of their skull divergence is due to directional selection linked with variation in temperature and precipitation seasonality (Simon, Machado, & Marroig, 2016). Given that we expect functional relations among skull traits to be important for population mean fitness, it follows that ecological factors exerting distinct selective pressures on the toad species skulls may induce some divergence in the relation between fitness and functional interactions among skull traits (Berg, 1960; Cheverud, 1984; Schwenk & Wagner, 2001). If divergent climatic conditions impose a different pattern of selection on the toad skull causing differences in the relation between functional interactions and fitness, we expect species from distinct climatic regimes to have higher differences in skull trait covariance pattern than species from similar climates. Therefore, we tested a second hypothesis that differences in skull integration pattern among the toad species are associated with variation in climatic regimes.

## 2 MATERIALS AND METHODS

### 2.1 Sample, 3D landmarking, and linear distances

We used a total of 1,064 specimens belonging to 10 species of the *R. granulosa* group (excluding *R. bernardoi* and *R. azarai* due to scarcity of specimens in museums) plus an out‐group species *R. margaritifera* (Table S1). We identified the species following the taxonomic units proposed by Narvaes & Rodrigues (2009). Although size might indicate the age of adult individuals, we could not determine age classes in our sample because there are no external features in toads that are correlated with age. Young juveniles are easy to distinguish from adults and were not used in this study. We scanned all specimens using an X‐ray microcomputed tomography system (micro‐CT, SkyScan 1176; Konitch, Belgium) at the Instituto de Biociências, Universidade de São Paulo, Brazil. We scanned specimens using the same resolution (pixel size = 18 μm), but total X‐ray energy differed among individuals depending on the thickness of the filter used. We had to use distinct filters while scanning because several specimens varied in bone density. We have tested for an effect of filter type in placing landmarks and estimating linear distances in toad specimens, and we concluded that deviations are in an acceptable range (Simon & Marroig, 2015). We performed the reconstruction process with the NRecon software (SkyScan; Konitch) with parameter values as described in Simon & Marroig (2015).

**Figure 1:**
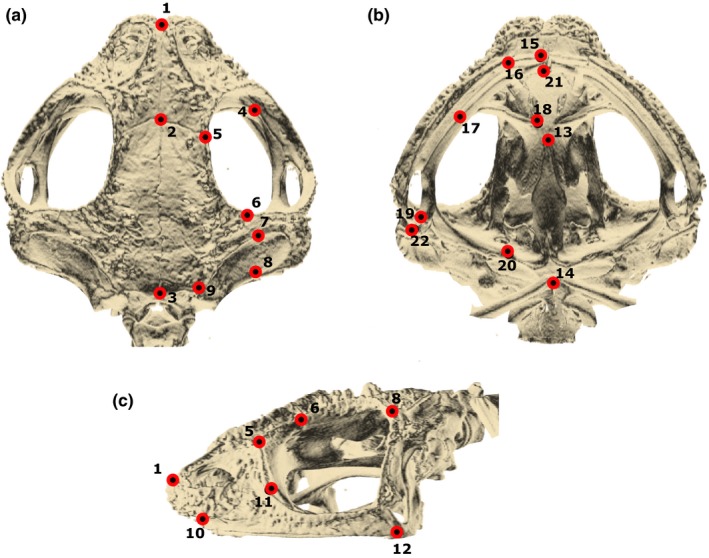
Numbered landmarks and linear distances in the toad skull. We placed 22 landmarks (red dots) spread in the whole skull: dorsal (a), ventral (b), and lateral (c) view. Landmarks are in bone sutures or bone processes to assure homology among species. Lines connecting the landmarks represent the 21 linear distances extracted from each specimen (see Table 1)

We placed 22 landmarks in each toad skull at bone sutures or bone processes (Figure 1, Table S2), so that we could assume their homology across all species. We used TINA Manual Landmarking Tool software (Schunke et al., 2012) to place landmarks in the 3D skull images. From these landmarks, we extracted 21 linear distances spread through the whole skull and allocated to specific developmental or functional units (Table 1). The distances are all individual bone dimensions thought to represent effectively heritable entities (Thomson, 1993) and to capture variation in local developmental and/or functional processes. As we have argued before (Simon & Marroig, 2015; Simon, Machado & Marroig, 2016), we prefer to use linear distances rather than landmark configurations to study morphological variation and covariation patterns because the superimposition procedure normally used to align specimens’ landmarks (the General Procrustes Analysis) may confound variation across landmarks (see van der Linde & Houle, 2009 and Marquez et al., 2012). Therefore, investigation of the biological causes of variation and covariation might be compromised when using covariance matrices based on Procrustes distances. Conversely, we are fully aware that using geometric morphometrics allows for a clear separation between scale (size), allometry, and shape (Zelditch, Swiderski, & Sheets, 1998) that would benefit our research. We tried to overcome this disadvantage by investigating separate effects of isometry and allometry on modularity (see below). We have carried out the landmarking process twice for each skull so we could detect and correct for gross measurement error and also calculate distance repeatability (Lessells & Boag, 1987), a measure of the proportion of variance among individuals not due to measurement error (Falconer & Mackay, 1996). Mean distance repeatability is 0.98 (range: 0.83 to 0.99). The distance with lower repeatability is in the premaxillary, which is the smallest distance (around 1.5 mm).

**Table 1:** Linear distances in the toad skulls and their allocation to the alternative modularity units

Distances	Landmarks	Bones	Developmental	Hormonal	Functional
1	1–2	Nasal	Hyoid (I, II, III)	T3++	Roof/snout
2	2–3	Frontoparietal	Hyoid (I, III)/mandibular (I, III)/branchial	T3+++	Roof/neurocranium/suspensorium II
3	1–4	Nasal	Hyoid (I, II, III)	T3++	Roof/snout
4	1–5	Nasal	Hyoid (I, II, III)	T3++	Roof/snout
5	5–6	Frontoparietal	Hyoid (I, III)/mandibular (I, III)/branchial	T3+++	Roof/neurocranium/suspensorium II/orbit
6	4–6	Orbit	–	–	Orbit
7	6–8	Squamosal	Mandibular (I, II, III)	T3++	Suspensorium (I, II)
8	7–9	Occipital	Branchial	T3+++	Roof
9	1–10	Prenasal	Hyoid (I, II, III)	T3++	Snout
10	1–11	Nasal	Hyoid (I, II, III)	T3++	Roof/snout
11	5–11	Nasal	Hyoid (I, II, III)	T3++	Roof/snout/orbit
12	10–12	Maxilla	Mandibular (I, II, III)	T3++	Snout
13	8–12	Squamosal	Mandibular (I, II, III)	T3++	Suspensorium (I, II)
14	13–14	Parasphenoid	Hyoid (I, II)/mandibular (I, II)	T3+++	Neurocranium
15	13–20	Parasphenoid	Hyoid (I, II)/mandibular (I, II)	T3+++	Neurocranium
16	15–16	Premaxilla	Hyoid (I, II)/mandibular (I, II)	T3++	Snout
17	16–17	Maxilla	Mandibular (I, II, III)	T3++	Snout
18	17–18	Neopalatine	–	T3+	Snout
19	17–19	Pterygoid	Mandibular (I, II, III)	T3+	Suspensorium (I, II)/orbit
20	19–20	Pterygoid	Mandibular (I, II, III)	T3+	Suspensorium (I, II)
21	21–22	Mandible	Mandibular (I, II, III)	T3+	Suspensorium (I, II)

Linear distances are all within single bones so that local variation of developmental and functional regulation factors may be captured. Developmental model is based on embryonic origin of the bones from three CNC streams. There are different configurations of the same developmental unit depending on which bones are assigned to each of them. Hormonal model is derived from differential sensitivity of toad skull bones to thyroxin hormone that triggers metamorphosis. Functional model is based on the division of the skull in five regions that perform specific functions.

### 2.2 Species phenotypic matrices

We represent integration/modularity patterns of the skull distances as pooled within‐species variance/covariance (V/CV) and Pearson product moment correlation phenotypic matrices (correlation). While correlation matrices are better suited to compare modularity patterns across species (because they are scale standardized), V/CV matrices are important for the study of evolutionary processes because they represent the variation pattern available for such processes to act upon. We performed outlier and normality analysis (Lilliefors’ test with significance level *p* < .05) of the distances in all species. Whenever we had adequate sample size, we constructed and compared within‐species P‐matrices for females and males separately to check whether the covariance and correlation patterns were stable despite a potential sexual dimorphism affecting the skull distances’ means. We did the same analysis also for within‐species locality‐specific P‐matrices. The lowest matrix similarity that we found was 0.83 using Random Skewers method (described below) between two locality‐specific P‐matrices for *R. mirandaribeiroi*. Thus, we conclude that species P‐matrices are stable enough in face of sexual and geographical variation. Still, when merging specimens of a given species of distinct sex and localities to estimate P‐matrices, these factors might promote correlations among skull distances that do not reflect the pleiotropic pattern of gene effects that we are interested in estimating (Porto et al., 2009), but instead, the simple differences in group means. Hence, we used multivariate linear models to remove significant sex and geography effects on skull distances’ means (tested with MANOVA and univariate analysis; Table S1). Therefore, species P‐matrices were constructed from the residuals of the appropriate linear models (using function “CalculateMatrix” from the “evolqg” R package; Melo et al. 2015). Given that V/CV and correlation P‐matrices are estimated with sampling error, we calculated matrix repeatability using 1,000 resampled P‐matrices for each species by applying a Monte Carlo resampling procedure (Manly, 2004). The distribution of resampled matrices was constructed using the empirical matrix and the species sample size as the parameters in a random multivariate normal (function “rmvnorm” from the “mvtnorm” R package; Genz et al., 2008). Matrix repeatability corresponds to the mean similarity by Random Skewers (see method description in section 2.4; Cheverud & Marroig, 2007) between each species empirical matrix with the 1,000 resampled matrices and indicates how reliable the empirical P‐matrices are.

### 2.3 Developmental versus functional modularity

Our first prediction is that the species P‐matrices have a modular correlation pattern among skull distances that resembles functional processes more than developmental ones. The first step to test this prediction is to analyze the evidence in favor of a modular signal of the developmental and functional units in the skulls of the toad species. We constructed three partial overlapping modularity models for the skull distances to inspect for modularity (Table 1). For the developmental model, we used Piekarski, Gross & Hanken, (2014) data on the contribution of three distinct CNC streams to the bony skull in *X. laevis*: (i) branchial, (ii) hyoid, and (iii) mandibular. The correspondence of individual bones to these CNC streams is not straightforward because the premaxillary, frontoparietal, and parasphenoid bones are derived from more than one stream. To accommodate this complexity in our modularity models, we tested alternative developmental units for a modular signal that differ on the assignment of these three bones (Table 1). Branchial, hyoid I, and mandibular I all have the frontoparietal bone, whereas hyoid II and mandibular II do not have it. Hyoid I and II as well as mandibular I and II have the premaxillary and parasphenoid bones, while hyoid III and mandibular III do not. We also tested different total modularity models, combining in different ways the developmental units, to investigate whether changing bone assignment affects the modular signal.

The functional model is based on anatomical subsystems that exert independent functional roles (Cheverud, 1982). The general functions of the bones are being sites for muscle attachment and promoting stability, protection, and support of soft tissues (Emerson, 1982; Kathe, 1999; Trueb, 1993). Bone sutures are related to bending strength and energy absorption of the skull (Kathe, 1999). We divided the skull into five functional units: (i) neurocranium: brain and auditory capsule protection and sound reception (Trueb, 1993); (ii) skull roof: protection against desiccation and/or predation by the behavior of phragmosis (hiding in holes and closing them with the dorsal region of the head, a common behavior of the *R. granulosa* species group; Gallardo 1965; Narvaes & Rodrigues, 2009; experimental evidence in other species: Jared et al., 2005; Navas, Jared, & Antoniazzi, 2002; Seibert, Lillywhite, & Wassersug, 1974); (iii) snout: detection of chemical signals by the vomeronasal organ (Halpern & Martinez‐Marcos, 2003) and of water‐borne cues by the olfactory epithelium, which is very developed in bufonids, especially species that bury themselves in the ground (Jungblut, Pozzi, & Paz, 2011; Sanuy & Joly, 2009); (iv) suspensorium: bones and insertion site of muscles (*M. submentalis* and jaw levators) that open the mandible for prey capture by tongue protraction (Haas, 2001; Nishikawa, 1999; Nishikawa & Gans, 1996); and (v) orbital region: visualization of prey size and prey movements (Nishikawa, 1999). We tested two alternative suspensorium units, with or without the frontoparietal (suspensorium II and I, respectively) that is a site of muscle attachment (Haas, 2001).

Even though we are primarily interested in comparing developmental against functional models, we also created a hormonal model to explore whether metamorphosis regulation might imprint a modular signal in the skull trait correlations. This model was based on information about thyroid hormone (T3) sensitivity in *Bombina orientalis* (Hanken & Hall, 1988a) and the temporal sequence of ossification in *Anaxyrus boreas* (former *Bufo boreas*; Gaudin, 1978) and *X. laevis* (Trueb & Hanken, 1992). We assumed that bones that ossify earlier are more sensitive to T3 than bones that ossify later during metamorphosis (Hanken & Hall, 1988b; Rose & Reiss, 1993). The first ossifying bones are the occipital, the frontoparietal, and the parasphenoid in *B. orientalis*,* A. boreas,* and *X. laevis* (Gaudin, 1978; Hanken & Hall, 1988a; Trueb & Hanken, 1992). Therefore, we constructed three units that potentially differ on T3 sensitivity for our toad species: (i) T3+++: bones that ossify first and are more sensitive to T3; (ii) T3++: bones that ossify in the middle of metamorphosis and have intermediate T3 sensitivity (iii) T3+: last ossifying bones and less sensitive to T3 (see Table 1). The three‐first ossifying bones are the same ones that compose the neurocranium, hence this unit may be considered as a functional–hormonal unit.

We then used the previously constructed distributions of 1,000 resampled P‐matrices for each species to test whether the empirical difference between the average trait correlations within the hypothetical modules (AVG+) and the average correlations between modules (AVG−) is different from zero (we call this difference “AVG diff”). We calculated 1,000 resampled AVG diff values for each hypothetical module to construct 95% confidence intervals (IC95) for the empirical AVG diff. We consider AVG diff as significant when the IC95 does not contain zero. Evidence for a modular pattern in the toad skulls consists of significant positive values of AVG diff because we expect AVG+ to be higher than AVG− and the higher is AVG diff, the higher is the degree of modularity. We tested three types of P‐matrices for modularity signal: with size variation and without allometric or isometric size variation. Throughout the development of an individual, growth effects are spread through all traits and may create high correlations between modules in addition to enhancing correlations within modules, therefore masking a potential modularity signal (Marroig, Vivo, & Cheverud, 2004; Mitteroecker & Bookstein, 2007). Whereas isometric growth affects all traits by the same scaling factor, allometric growth changes some traits more than others producing shape changes that may have functional relevance (Young & Hallgrímsson, 2005). If faster growth of a set of traits in relation to the total body growth promotes higher correlations within this set than between ‐sets, removing allometric size variation may actually erase some modularity signal. Hence, we removed size variation from the species P‐matrices before testing them against the different modularity models in two different ways: (i) removing just isometric variation or (ii) removing allometric size variation. Isometric variation was removed by following Somers (1989) procedure to calculate log‐transformed double‐centered data (see Supporting Information for more details). Allometric variation was removed by subtracting variation associated to the first principal component (PC1) of the V/CV matrices and then transforming them into correlation matrices (similar to Marroig et al., 2004; Porto et al., 2013). PC1 in all species can be interpreted as allometric size because all its coefficients have the same sign but are not all equal in log scale (Jolicoeur, 1963).

The second step in the modularity analysis was to construct modularity models composed of modules that have empirical evidence and compare the empirical support for each of them. There are currently two modularity analyses that allow for the direct comparison of alternative modularity models: Márquez (2008) analysis that models covariance matrices as the outcome of spatially overlapping modular effects, and Goswami and Finarelli (2016) analysis that compares likelihood surfaces of modularity models with variable complexity (“EMMLi” R package). We chose to use Goswami and Finarelli (2016) method because the distribution of trait correlations is explicitly modeled (normal distribution of Fisher r‐z‐transformed correlations); the intermodule correlations are not determined as zero, and models are compared by AICc values to determine which model has the strongest support from empirical data. Yet, a drawback of using this analysis is that we cannot assign a single linear distance (or landmark) to more than one module. This becomes a problem when there are traits such as the frontoparietal bone that belongs to more than one developmental and functional modules. Thus, we had to construct alternative modularity models that differ in the assignment of some bones to specific modules. We labeled the alternative models as “a” and “b”, and we use an asterisk mark alongside modules that had some bones retrieved from its original composition (see Table 2). In the case of the branchial unit, it could only be tested with the frontoparietal because it is composed of just the frontoparietal and the occipital. Thus, we tested developmental models with and without the branchial unit (in this last case, the frontoparietal can belong to the hyoid I and III or the mandibular I and III). We did not test all functional units in the same model because the neurocranium would end up with just the parasphenoid bone (frontoparietal and occipital would be in the roof unit). Instead, we compared models with the neurocranium or with the roof and with all other functional units. We directly compared modularity models to P‐matrices with size variation as well as without size variation. By removing size variation from the P‐matrices, several correlations that were positive became negative (see Fig. S2); therefore, we used the argument “abs” = false in the “EMMLi” R function that determines whether estimated correlations (“rho”) will be absolute or not, therefore, allowing that negative values for the correlations could be estimated. We followed Goswami and Finarelli (2016) recommendation of checking the posterior probability of the best‐supported models (probability of the model in relation to all other models tested, varying from 0.0 to 1.0), and we considered the results unreliable if the posterior probability of the model is <0.5.

**Table 2:** Alternative modularity models composed of different modular units

Models	With size variation
Developmental Ia	Hyoid II*, mandibular II
Developmental Ib	Hyoid II, mandibular II*
Hormonal I	T3+, T3++
Functional I	Snout, suspensorium I

	No allometric Variation
Developmental II	Branchial, hyoid II
Hormonal II	T3++, T3+++
Functional II	Neurocranium*, snout, orbit, suspensorium I
Functional IIIa	Roof, snout*, orbit, suspensorium I
Functional IIIb	Roof*, snout, orbit, suspensorium I
Functional IV	Neurocranium*, snout, orbit
Functional Va	Roof, snout*, orbit
Functional Vb	Roof*, snout, orbit
Functional VI	Snout, orbit, suspensorium I

	No isometric variation
Developmental II	Branchial, hyoid II
Developmental III	Branchial, mandibular II
Developmental IV	Branchial, hyoid II, mandibular III**
Developmental V	Branchial, hyoid III**, mandibular II
Developmental VI	Hyoid II, mandibular III
Developmental VII	Hyoid III, mandibular II
Hormonal I	T3+, T3++
Hormonal II	T3++, T3+++
Hormonal III	T3+, T3++, T3+++
Functional II	Neurocranium*, snout, orbit, suspensorium I
Functional IIIa	Roof snout*, orbit, suspensorium I
Functional IIIb	Roof*, snout, orbit, suspensorium I
Functional IV	Neurocranium*, snout, orbit
Functional Va	Roof*, snout, orbit
Functional Vb	Roof, snout*, orbit
Functional VI	Snout, orbit, suspensorium I
Functional VII	Neurocranium**, snout, orbit, suspensorium II
Functional VIII	Snout, orbit, suspensorium II

Models are only composed of developmental, hormonal, or functional units that had a modular signal depending on the type of P‐matrix. Hyoid II* and mandibular II* do not have premaxillary and parasphenoid bones. Neurocranium* does not have one of the frontoparietal distances. Neurocranium** does not have both frontoparietal distances. Roof* and snout* do not have most nasal distances. Mandibular III* and hyoid III* do not have the frontoparietal bone.

### 2.4 Roles of phylogeny and climate on P‐matrix dissimilarity

In order to test our second prediction that differences in skull trait covariance are associated to variation in climatic conditions across species, we constructed a variation partitioning model (Desdevises et al., 2003; function “var.part” from “vegan” R package Oksanen et al., 2008) to compute the contribution of both phylogeny and climate to the divergence in species V/CV P‐matrices. We chose this particular analysis because it estimates the independent contributions of phylogeny and climate in explaining species P‐matrix divergence, but it also calculates the so‐called “phylogenetically structured environmental variation” (Desdevises et al., 2003), that is, potential differences in V/CV P‐matrices explained by climatic variation correlated with phylogeny. We considered climatic variation correlated with phylogeny to be important because we detected a significant phylogenetic signal in the species mean climatic variables (*K*_mult_ = 1.34; *p* = .02; using function “physignal” of the “geomorph” package, Adams, 2014), and this specific variation component may be relevant in producing P‐matrix differences. A phylogenetic signal above 1.0 indicates that climatic regimes of closely related species are more similar than expected by the Brownian motion model (Blomberg, Garland, & Ives, 2003). We preferred to use variation partitioning instead of traditional comparative methods (CM), such as phylogenetic least‐square regression (PGLS; Grafen, 1989), because CM allocate the maximum portion of variation in the dependent variable to phylogeny and leaves only the residual variance to be tested for ecological effects (Westoby, Leishman, & Lord, 1995). Therefore, not finding a significant effect of ecology on some variable using CM might be a real biological phenomenon or an artifact of the analysis (which is explicitly accounted for in variation partitioning analysis). However, a drawback of not using CM is to not have an explicit evolutionary model underlying P‐matrix evolution in the analysis.

To construct the variation partitioning models, we first created a dissimilarity matrix used as the dependent variable in the models. We calculated similarity indexes among all species V/CV P‐matrices using Random Skewers analysis (RS; Cheverud & Marroig, 2005). RS is directly derived from Lande's (1979) multivariate selection equation (Δ*z* = G β), in which a 1,000 random selection vectors (β) are applied to a pair of species matrices being compared and the overall similarity index (S) is the average correlation between the species responses (Δ*z*) to the same selection. To transform similarity values into dissimilarity ones, we computed the squared root of (1 – S) for each pairwise comparison (Legendre & Legendre, 1998). Therefore, high values in the dissimilarity matrix indicate high divergence in the response to selection between a pair of species, whereas low values indicate low divergence in species response to selection. We constructed two dissimilarity matrices: one with raw P‐matrices and another with residual P‐matrices in which isometric size variation was removed. As the dissimilarity matrix has values that are not independent from each other, we performed a principal coordinate analysis (PCoA) to extract ordination axes in which we projected distances among the P‐matrices (Mitteroecker & Bookstein, 2009; Legendre & Legendre, 1998). PCoA produces a representation of the objects to be compared in an Euclidian space for which the relations among objects is preserved (Legendre & Legendre, 1998) and also makes the visualization of the differences in P‐matrices more evident (Mitteroecker & Bookstein, 2009). There are other methods to compare covariance matrices with different null hypothesis and biological meanings (e.g., Arnold & Phillips, 1999; Calsbeek & Goodnight, 2009) or distance‐based methods (Mitteroecker & Bookstein, 2009). Mitteroecker & Bookstein (2009) argued that a metric using Relative Eigenanalysis (RE) provides a natural distance between covariance matrices when considering developmental factors that can also be represented in an ordination space. Thus, given that RS and RE focus on different aspects of the P‐matrices, we also constructed variation partitioning models with PCoA from Mitteroecker & Bookstein (2009) distance matrix (square root of the sum of the squared log‐transformed relative eigenvalues between two covariance matrices). We corrected the P‐matrices for noise before performing RE using the method described in Marroig, Melo, & Garcia, (2012) because the P‐matrices are inverted in this analysis.

The two independent factors in the variation partitioning models are phylogeny and climate. The phylogeny is represented by PCo axes (as in Desdevises et al., 2003; following Diniz‐Filho, de Sant'Ana, & Bini, 1998) derived from a phylogenetic distance matrix (function “cophenetic.phylo” from “phytools” R package; Revell, 2012; Table S3) calculated from a Bayesian molecular phylogeny (Pereyra et al., 2015). For the climatic data, we used the BioClim database (Busby, 1991) composed of 17 variables (we excluded BIO3 and BIO7, both related to thermal amplitude, because they are linear combinations of other variables) calculated from monthly precipitation (mm) and temperature (°C) records obtained from several climatic stations (Hijmans et al., 2005; spatial resolution of approximately 1 km^2^). We extracted the climatic data from all localities where species from the *R. granulosa* complex are reported to occur (Narvaes, 2003) using DIVA‐GIS software. Given that temperature and precipitation data have very different scales (°C and mm) and that variances of precipitation data are much higher than temperature data, we opted to transform all climatic data using the *z*‐score transformation (e.g., Duran & Pie, 2015) before constructing a climatic correlation matrix and extracting its principal components (PCs; Table S4). We followed Desdevises et al. (2003) to decide which PCo axes of the V/CV P‐matrix dissimilarity were the dependent variables: only the ones for which there were significant correlations with any independent factor. Likewise, the phylogenetic PCo axes and climatic PCs used as the independent factors in the variation partitioning models were only those that had a significant correlation with the V/CV P‐matrix dissimilarity axes. We present the adjusted variation in P‐matrix dissimilarity explained by phylogeny and climate as well as their significance using redundancy analysis (function “rda” from “vegan” R package; Oksanen et al., 2008).

## 3 RESULTS

### 3.1 Phenotypic trait correlations and size variation

The toad species V/CV and correlation P‐matrices have high repeatability values (mean = 0.97 ± 0.015 and mean = 0.98 ± 0.014, respectively) indicating that sampling error is low. Skull distance correlations are practically all positive and have an average of 0.68 ± 0.13 for the species of the *R. granulosa* complex, being lower only for the external group species *R. margaritifera* (average = 0.4 ± 0.1; see Fig. S1). All the species from the *R. granulosa* complex have a high percentage of total variance due to size variation, isometric (57% to 81%) or allometric (64% to 84%), being a little lower only in the external group, *R. margaritifera* (51% and 36% respectively; see Table S5).

### 3.2 Toad skull modularity

**Figure 2:**
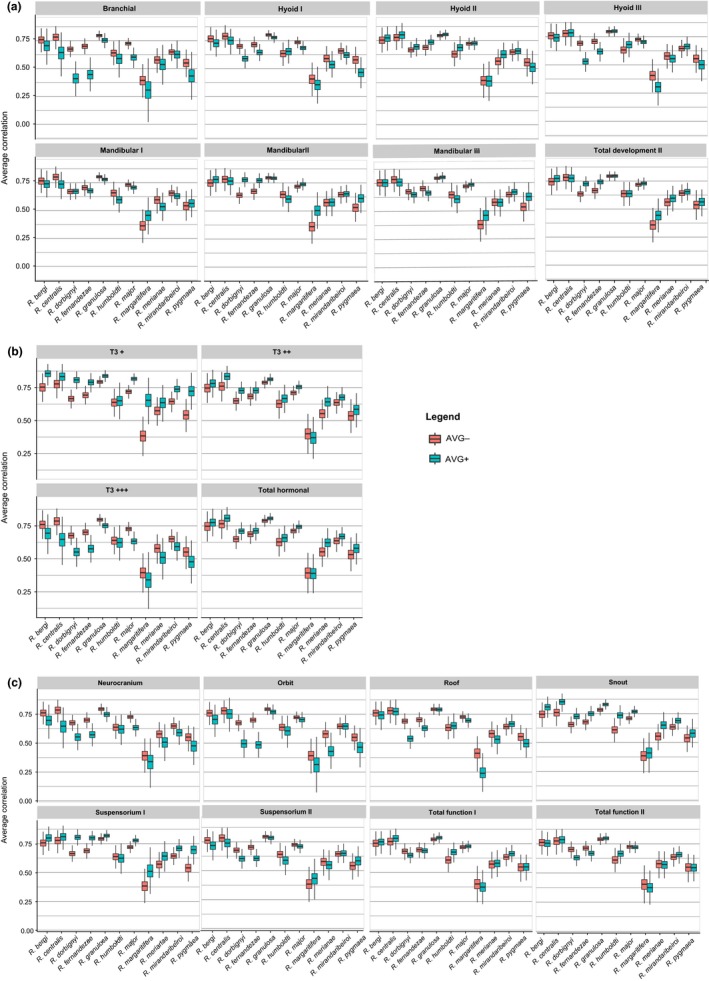
Average correlations within (AVG+) and between (AVG−) hypothetical modules for P‐matrices with size variation. We performed a Monte Carlo resampling procedure on the species data to construct 1,000 resampled P‐matrices for each species and 95% confidence intervals for the difference between AVG+ and AVG−. AVG+ are blue box plots, whereas AVG− are red box plots. Significant differences in AVG+ and AVG− are indicated with an asterisk. (a) Developmental model: We tested alternative hyoid and mandibular units that differ on the assignment of specific bones. Total Development II is composed of branchial, hyoid II, and mandibular II units. (b) Hormonal model: Total Hormonal is composed of all three units. (c) Functional model: We tested alternative suspensorium units. We present both Total Function I and Total Function II that are composed of all five units, differing on the alternative suspensorium unit (I or II, respectively)

Hyoid II and mandibular II developmental units are supported (positive significant AVG diff values) in a few of the species P‐matrices with size variation (Figure 2a, Table S6). Practically, all species have the hormonal units T3+ and T3++ (as well as Total Hormonal; Figure 2b, Table S7) and the functional units snout and suspensorium I (Figure 2c, Table S8). Based on these results, we compared the ML support for modularity models composed of just these units (see Table 3). Functional I (snout, suspensorium I) and Hormonal I (T3+, T3++) are the preferred models in four species each, whereas Developmental Ib (hyoid II, mandibular II*) is best supported in two species (Table 3). Yet, the posterior probabilities of all of the preferred models are low (equal to or below 0.5), indicating that the ML analysis did not perform well in these cases.

**Table 3:** Preferred modularity models for the toad species skull correlation pattern

Species	Model (with size)	MaxL	K	AICc	Post_Prob
*R. centralis*	Functional I	137.9	5	−265.4	0.40
*R. humboldti*	Functional I	113.5	4	−218.9	0.55
*R. merianae*	Functional I	87.3	4	−166.4	0.32
*R. granulosa*	Functional I	−194.2	5	398.6	0.37
*R. mirandaribeiroi*	Hormonal I	35.6	5	−61.0	0.36
*R. major*	Hormonal I	−374.2	5	758.6	0.50
*R. bergi*	Hormonal I	92.0	5	−173.8	0.44
*R. pygmaea*	Developmental Ib | Hormonal I	78.6 | 77.6	5 | 5	−146.9 | −144.9	0.4 | 0.12
*R. dorbignyi*	Developmental Ib	−410.5	5	831.2	0.50
*R. fernandezae*	Developmental Ib	−503.1	5	1016.6	0.50
*R. margaritifera*	Hormonal I	173.9	5	−337.6	0.50

	Model (no allometric size)				
*R. centralis*	Functional IIIb	76.10	7	−137.70	0.68
*R. humboldti*	Functional II	93.0	11	−162.6	0.89
*R. merianae*	Developmental II	24.30	5	−38.20	0.46
*R. granulosa*	Functional II	−276.60	11	576.50	0.94
*R. mirandaribeiroi*	Functional IV | II	−85.5 | −82.9	8 | 11	187.7 | 189.1	0.35 | 0.17
*R. major*	Functional II	−225	11	473.4	1.0
*R. bergi*	Developmental II	109.6	4	−211	0.39
*R. pygmaea*	Functional II | IIIb | IV	90.2 | 85.1 | 87.2	8 | 4 | 6	−163.8 | −162.0 | −162.0	0.25 | 0.10 | 0.10
*R. dorbignyi*	Functional II	45.1	11	−66.8	0.76
*R. fernandezae*	Functional II	−197	11	417.4	0.92
*R. margaritifera*	Functional II | IIIb	108.8 | 104.0	8 | 4	−201.0 | −199.9	0.24 | 0.14

	Model (no isometric size)				
*R. centralis*	Functional VII	80.2	6	−148.1	0.25
*R. humboldti*	Functional VII	134.4	11	−245.4	0.96
*R. merianae*	Developmental IV	55.4	8	−94	0.86
*R. granulosa*	Functional VII	−227.5	11	478.3	1.0
*R. mirandaribeiroi*	Functional II | VII | IV	−81.8 | −82.4 | −85.7	11| 11 | 8	186.9 | 188.1 | 188.2	0.48 | 0.27 | 0.25
*R. major*	Functional VII	−184.3	11	391.9	0.99
*R. bergi*	Functional VII	98.4	11	−173.6	0.85
*R. pygmaea*	Developmental V | Functional VII	123.1 | 125.4	8 | 11	−229.4 | −227.6	0.34 | 0.13
*R. dorbignyi*	Hormonal III	22.5	8	−28.4	0.97
*R. fernandezae*	Developmental IV	−245.6	8	508.0	0.87
*R. margaritifera*	Developmental IV	130.9	8	−245.1	0.78

We compared the support for different modularity models using maximum likelihood. Results are shown for P‐matrices with size variation and for residual P‐matrices with no allometric or isometric size variation.

MaxL, maximum likelihood; K, number of estimated mean correlations; AICc, Akaike information criterion for small samples sizes; Post_Prob, posterior probability of the models.

**Figure 3:**
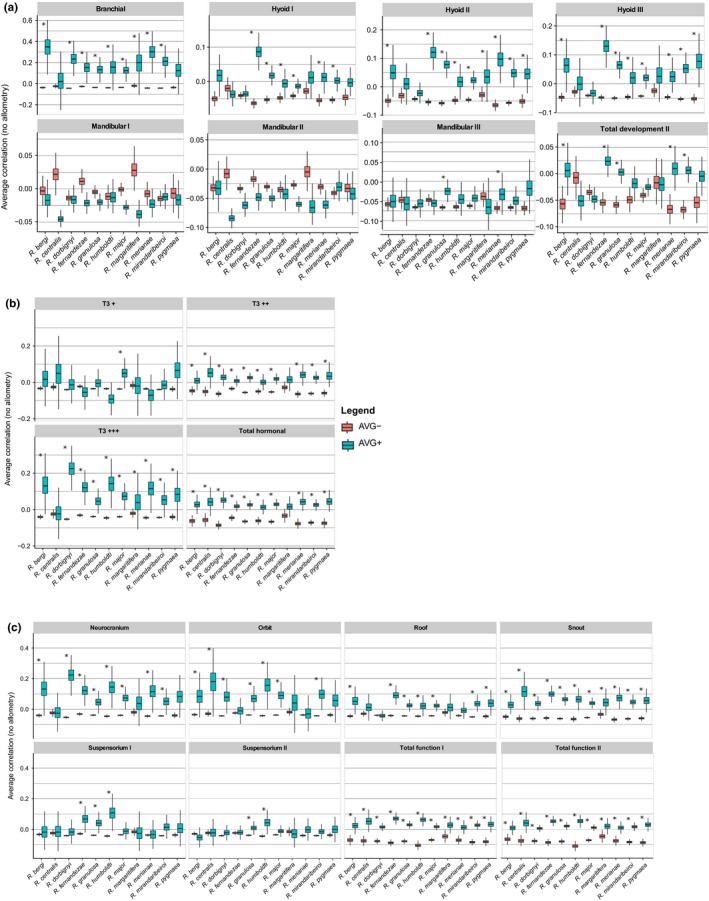
Average residual correlations within (AVG+) and between (AVG−) hypothetical modules for P‐matrices without allometric size variation. AVG+ are blue box plots, AVG− are red box plots, and asterisks indicate significant difference between them. (a) Developmental model: We tested the same alternative units as described in legend of Figure 2a. (b) Hormonal model: We tested the same alternative units as described in legend of Figure 2b. (c) Functional model: We tested the same alternative units as described in legend of Figure 2c

When allometric size variation was removed, we can notice that the branchial, hyoid II, and III developmental units have a modular signal in practically all the toad species and hyoid I in six species (Figure 3a, Table S9). The T3+++ hormonal unit also appears in all species (except *R. centralis*), along with T3++ and Total Hormonal (Figure 3b, Table S10); as well as the neurocranium (except *R. centralis* once again) and snout functional units, along with Total Function I and II (Figure 3c, Table S11). Orbit and roof units have a modular signal in seven species. In contrast, very few species have support for the mandibular and suspensorium units when allometry is removed. AVG diff values are higher in more species when allometric size variation was removed compared to P‐matrices with size variation (see Tables S9–S11). Functional II (neurocranium*, snout, orbit, and suspensorium I) or/and Functional IIIb (roof*, snout, orbit, and suspensorium I) modularity models have higher support in nine species, while Developmental II (branchial and hyoid II) is the preferred model in two species (Table 3). Yet, only functional models have high posterior probabilities.

**Figure 4:**
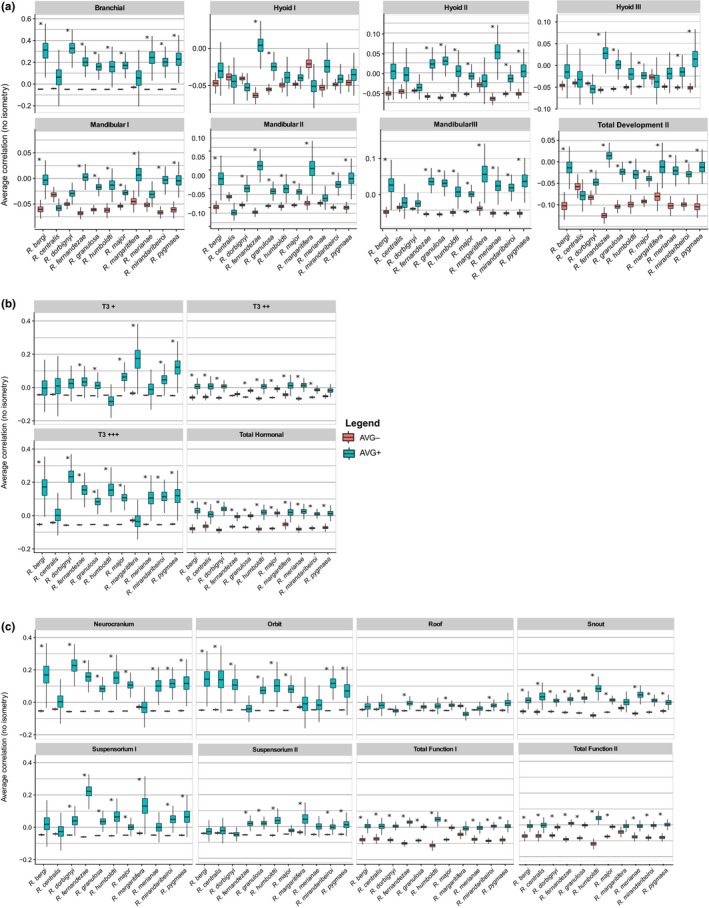
Average residual correlations within (AVG+) and between (AVG−) hypothetical modules for P‐matrices without size isometric variation. AVG+ are blue box plots, AVG− are red box plots, and asterisks indicate significant difference between them. (a) Developmental model. (b) Hormonal model. (c) Functional model

For P‐matrices without isometric size variation, mandibular I, II, and III developmental units, suspensorium I and II functional units and the T3+ hormonal unit also show modular signal in at least six species up to all species (Figure 4, Tables S12–S14). On the other hand, the roof functional unit ceases to have a modular signal in most species. Functional VII (neurocranium**, snout, orbit, and suspensorium II) modularity model is the preferred one in six species when isometric size variation was removed (four species with high posterior probability; Table 3). Developmental IV model (branchial, hyoid II, and mandibular III**) has the highest support in three species (all with high posterior probabilities). Just *R. dorbignyi* has a hormonal model as the preferred one (Hormonal III: T3+, T3++, T3+++; Table 3).

### 3.3 Climatic variation partially explains P‐matrix dissimilarity

**Figure 5:**
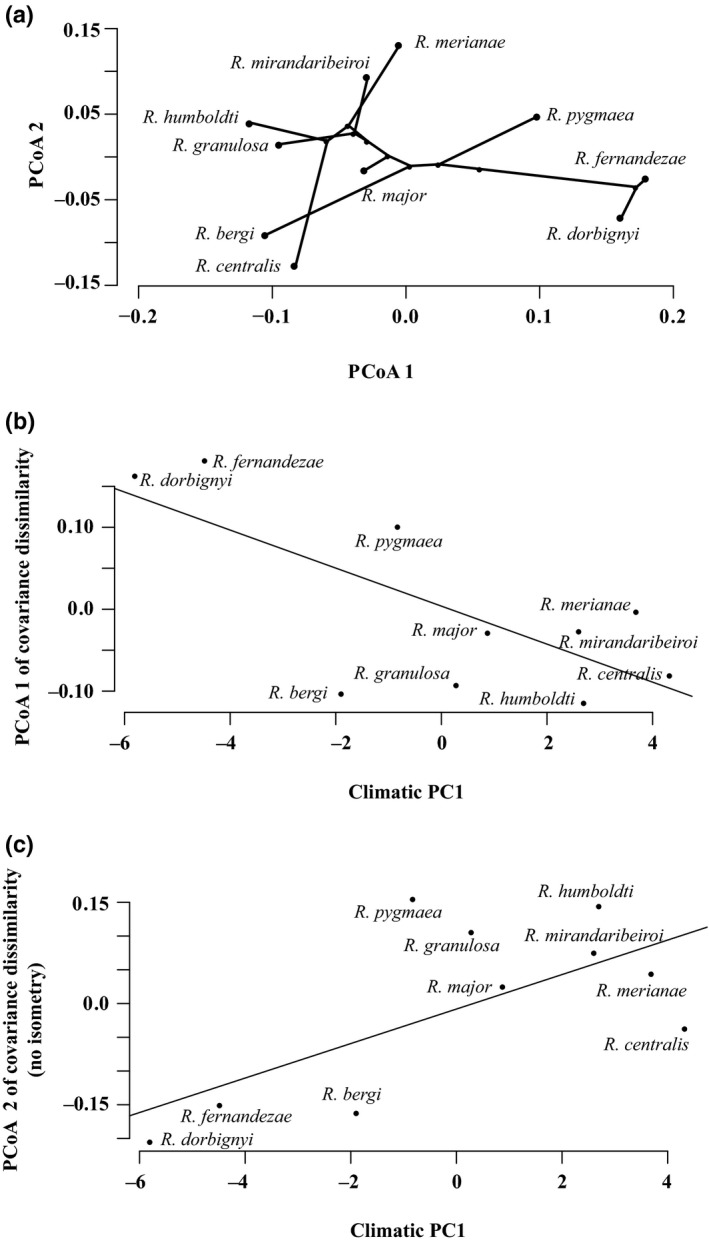
Principal coordinate (PCo) axes for P‐matrix dissimilarity and relations with phylogeny and climatic variation. (a) The plot shows how much species covariance P‐matrices differ in an ordination space constructed from PCo axes 1 and 2 of a dissimilarity matrix in which P‐matrices were compared by Random Skewers and their relation with phylogeny. (b) Association between variation in P‐matrix dissimilarity and species scores projected on the first principal component (PC1) of the climatic matrix. Species that differ less in their P‐matrices have more similar climatic regimes. (c) Species that differ less in P‐matrices still are the ones with more similar climatic regimes even when isometric size variation was removed

Species P‐matrices are more dissimilar when isometric size variation is removed (Table S15). The variation partitioning model for P‐matrix dissimilarity using RS was constructed with only the first PCo axis because it is the only one that correlates significantly with the independent factors (cor = .75, *p* = .02 with PCoA 1 of phylogenetic distance; cor = −.72, *p* = .02 with climatic PC1; see Figure 5a,b). The first PCo axis of the dissimilarity matrix separates the three most external species in the phylogeny (*R. dorbignyi* + *R. fernandezae*) + *R. pygmaea* from the rest of the species (Figure 5a). Phylogeny independent of climate does not explain any variation in P‐matrix dissimilarity, and the same occurs for climate independent of phylogeny (Table 4). Therefore, all variation in P‐matrix dissimilarity accounted for PCoA 1 explained by phylogeny and climate (15.2%) corresponds to the phylogenetically structured climatic variation (Table 4). *R. dorbignyi* and *R*. *fernandezae*, the most basal species in the phylogeny and the ones subjected to much more temperature seasonality, lower mean temperatures, and lower mean precipitation in the wettest months (see Table S9), have the most similar P‐matrices and differ the most from *R. centralis*,* R. merianae,* and *R. humboldti*, which are subjected to the opposite climatic pattern (Figure 5b, Table S18). Phylogenetically structured climatic variation is also relevant to explain P‐matrix dissimilarity when using RE (see Tables S16 and S17, Fig. S3). PCoA 2 of the dissimilarity matrix using RE has a correlation of 0.8 (*p* = .02) with PCoA 1 of the dissimilarity matrix using RS.

**Table 4:** Variation partitioning models for P‐matrix dissimilarity

PCoA covariance dissimilarity			
	Adjusted *R* ^2^	*F*	*p*
With size			
Phylogeny	**0.5**	**10.0**	**.025**
Climate	**0.45**	**8.47**	**.02**
Phylogeny | Climate	−0.01	0.83	.4
Climate | Phylogeny	−0.06	0.16	.7
Phylogeny: Climate	0.51	–	–
No isometric size			
Phylogeny	0.19	3.21	.12
Climate	**0.23**	**3.7**	**.02**
Phylogeny | Climate	0.25	4.9	.07
Climate | Phylogeny	**0.29**	**5.48**	**.015**
Phylogeny: Climate	−0.06	–	–

The dependant variables in the model are principal coordinate axes (PCoAs 1 and 2) of the dissimilarity among species P‐matrices measured with Random Skewers. The independent variables are the PCoA 1 or 7 of the phylogenetic distance matrix and species scores on climatic PC1. We ran the analysis with raw P‐matrices and with P‐matrices for which isometric size was removed. Phylogeny | Climate is the variation in dissimilarity explained by phylogeny independent of climate, whereas Climate | Phylogeny is the variation explained by climate independent of phylogeny. Phylogeny: Climate corresponds to the phylogenetically structured climatic variation (see text). The variation in P‐matrix dissimilarity explained by shared effects of climate and phylogeny can be calculated but not statistically tested because there are no degrees of freedom associated with it. Values in bold are significant at P < 0.05 using redundancy analysis.

When isometric size variation was removed from the species P‐matrices, a different variation partitioning model was constructed with PCoA 1 and PCoA 2 as the dependent variables that have significant correlations with the independent factors (cor = .69, *p* = .03: PCoA 1 of P‐matrix dissimilarity with PCoA 7 of phylogenetic distance; cor = .66, *p* = .04: PCoA 2 of P‐matrix dissimilarity with climatic PC1; Figure 3c). Climatic variation among species independent of phylogeny explains part of the variation in P‐matrix dissimilarity (11.0%; Table 4). For the model using RE, the removal of isometric size variation did not change the relevance of the phylogenetic structured climatic variation in explaining variation in P‐matrix dissimilarity (Table S17, Fig. S3).

## 4 DISCUSSION

### 4.1 Do functional interactions among traits influence the adaptive landscape?

Functional morphologists and ecologists as well as evolutionary biologists widely acknowledge that variation in mean morphology may lead to variation in functional performance, which on its turn may lead to variation in fitness (Arnold, 1983; Losos, 1990; Wainwright, 2007). That is, it is well accepted that mean morphologies of natural populations might evolve through directional selection related to differences among individuals in function. Nevertheless, how much functional interactions among traits actually influence fitness and shape patterns of stabilizing selection acting on trait covariances and correlations is still poorly understood (Arnold, 2005; Klingenberg et al., 2010; Young & Badyaev, 2006; Zelditch & Swiderski, 2011). We studied variational modularity and its relation to development and function to infer whether functional interactions among traits are important to population fitness. A functional modularity signal at the population level suggests that specific functional interactions are relevant to mean fitness and capable of molding development as a response to stabilizing selection acting on genetic pleiotropic effects (Cheverud, 1984; Cheverud 1996; Lande, 1980).

Given the occurrence of metamorphosis in anuran skull development, we expected to infer a higher contribution of functional interactions to adult skull trait correlations than of developmental interactions. After metamorphosis, the anuran skull starts to perform new functions related to terrestrial lifestyle and proper interactions among skull traits to perform these functions probably influence individual fitness in adults. Functional modularity overcame developmental modularity as we predicted, but only when size variation was maintained as part of the trait correlations. Functional modules were conspicuous in practically all species whereas developmental ones in just a few species (Figure 2). Removal of the frontoparietal from the mandibular and hyoid developmental units (mandibular II and hyoid II) improved its modular signal in some species compared to the same units with the frontoparietal (Figure 2a). Adding the frontoparietal to the suspensorium unit (suspensorium II) caused a loss of modular signal compared to suspensorium I (Figure 2c). Therefore, assignment of the frontoparietal bone to a specific unit weakens its modularity signal.

Hormonal‐regulated modules were also found in all species with size variation, indicating that events regulated by the thyroxin hormone during metamorphosis do contribute to skull trait correlation pattern. Detecting functional and hormonal‐related modules even when a global integrating factor such as size variation is present indicates that local functional and hormonal‐regulated processes have a strong influence in some bones, enhancing their correlations above the overall level of integration promoted by growth. Thus, the functional relations between bones composing the snout and suspensorium I modules, as well as relations among bones moderately and less sensitive to T3 hormonal regulation, are probably relevant for the species mean fitness, being subjected to stabilizing selection. The stabilizing selection may be either internal selection, related to proper control of metamorphosis and proper functioning; or external selection related to the interaction of the modules with the environment, or both. Yet, when correlations among traits are high, the ML analysis performs poorly (Finarelli & Goswami, 2016), as confirmed by the low posterior probabilities of modularity models with size variation (Table 3). The toad skull is a very integrated structure (as the high contribution of size variation to total phenotypic variation indicates; Table S5), and the degree of modularity seen in species P‐matrices with size variation is quite low (AVG diff values generally below 0.1). It is hard to pinpoint how large has to be the difference between average correlations within and between units so we may consider it biologically relevant and expect relative independent evolutionary responses from modules. Nevertheless, we have shown that this group of tropical toad species suffered high evolutionary constraints on the response to directional selection when studying the divergence of skull phenotypic means (Simon, Machado & Marroig, 2016).

When allometric or isometric size variation is removed from species P‐matrices, more units have a modular signal and the degree of modularity is higher (AVG diff higher than 0.1), independent of whether they are developmental, hormonal, or functional units (Figures 3 and 4). These results suggest that most modular units in the toad skull are masked by the presence of global integration factors, such as juvenile and adult growth that initiate only after metamorphosis. Hence, most Local processes promoting integration are probably not strong enough to enhance the correlations among bones above the overall influence of allometric or isometric growth. The suspensorium I and II functional modules, and the mandibular I and III developmental modules are conspicuous only when isometric size variation is removed (Figure 4a,c). This means that allometric growth effects influence the most the mandibular and suspensorium units that grow faster in relation to body growth (positive allometry; see Table S19) when compared to the rest of the skull, having their correlations enhanced by allometric growth. The species that diverged the most in its modularity pattern without size variation was *R. centralis*, not having the mandibular developmental units, nor the T3+++ hormonal unit and the neurocranium functional unit (also the case of *R. margaritifera*). Yet, these species are the ones with the lower sample size (*n* = 37 and 38, respectively) and one potential caveat is that their correlation pattern may not be as well estimated as the other species.

Thus, without the overriding effect of growth, we may infer that the skulls of the toad species are subjected to stabilizing selection related to development, hormonal sensitivity, and function, given that average correlations within modules are higher than average correlations between modules (Figures 3 and 4). However, only functional models are best supported using ML analysis when allometric size variation was removed (Table 3). This apparent discrepancy in results shows that a preferred modularity model does not exclude the existence of other modules in the organisms belonging to a nonpreferred modularity model. Thus, we recommend caution when interpreting model selection for modularity. Still, for P‐matrices without allometry, we may also infer that functional modularity overcomes developmental modularity, that is, the toad skull trait correlations have an organization that reflects more functional modules than developmental ones (even though we do detect developmental and hormonal‐regulated modules). Therefore, we may interpret our modularity results aligned with the Palimpsest model (Hallgrímsson et al., 2009), but not because metamorphosis erases the developmental modularity signal as we hypothesized, but because growth, being a process that initiates later in toad skull ontogeny and has a very extended duration (specially in species with indeterminate growth), blurs the hallmarks of earlier ontogenetic events, such as the embryonic origin of bones.

When isometric size variation is removed from the P‐matrices, species differ in which modularity model is best supported by empirical data. The toad species with the functional model as the best supported have the neurocranium, snout, orbital region, and suspensorium II units. In this functional model, one of the distances of the frontoparietal bone is assigned to the suspensorium II and the other to the orbital region. This means that the neurocranium is left with only the occipital and parasphenoid bones that protect mainly the ventral part of the skull. The neurocranium unit is the one with highest integration in the functional model, followed by the orbital region (Table S20). The strength of the stabilizing selection is proportional to the tightness of the fit between two characters when they interact (Pélabon, Armbruster, & Hansen, 2011; Schwenk & Wagner, 2001). Therefore, we suggest that neurocranial and orbital units are potentially subjected to stronger stabilizing selection than the snout and suspensorium units. The species in which the developmental model is the preferred one have all three developmental units, with the frontoparietal bone assigned to the branchial stream. Models that have the frontoparietal assigned to the hyoid or the mandibular streams do not have empirical support. Hence, even though the frontoparietal is derived from all three streams, it might have stronger developmental interactions with the occipital bone than with any other bone, possibly by tissue–tissue direct interactions. The three species in which development is the main process shaping skull trait correlations, *R. merianae*,* R. fernandezae,* and *R. margaritifera*, do not present the orbital region as a module (Figure 4c). The orbital unit is related to prey visualization and detection of differences in prey size. Depending on the size of the prey, toads will use tongue or jaw prehension (Nishikawa, 1999). But species may diverge on this ability and we need experimental tests to confirm that this is the case with the species of the *R. granulosa* complex.

Other empirical studies have shown an important contribution of functional interactions in shaping integration patterns in systems where functional and developmental models can also be separated, such as the mammal mandible (e.g., Monteiro, Bonato, & Dos Reis, 2005; Monteiro & Nogueira, 2010; Young & Badyaev, 2006; Zelditch, Wood, Bonett, & Swiderski, 2008). However, these studies differ on how much the correlation pattern is also consistent with developmental expectations. For instance, Monteiro, Bonato & Dos Reis (2005) found a high support for a developmental model tested in the mandible of rodent species; however, differences in integration patterns were associated to divergent functional demands (arboreal vs. fossorial species). Similarly, differences in functional demands among species were suggested by Monteiro & Nogueira (2010) when studying mandibular integration in phyllostomid bats. Perhaps one of the stronger examples of functional demands promoting changes in covariance structure is the forelimbs of some mammalian species, such as gibbons and bats, which have specialized functions and less covariation between fore‐ and hindlimbs than quadrupeds that share the same function for both limbs (Young & Hallgrímsson, 2005). In contrast, the cricket wing is an example of a structure that reflects developmental integration, responding as a single cohesive unit to selection, and not as separate functional units related to sound production (Klingenberg et al., 2010). Therefore, the empirical evidence so far, including our study, suggests that both developmental and functional interactions among traits influence the fitness surface, but divergence in phenotypic modularity patterns associated to function across species may depend on the strength of functional demands, and therefore the strength of stabilizing selection related to function, and on the influence of overall integration factors, such as growth.

### 4.2 Climate and skull morphological integration

The climatic variation that is structured by phylogeny explains part of the divergence in skull trait covariance pattern among the toad species. This suggests the occurrence of phylogenetic niche conservatism (Grafen, 1989; Westoby, Leishman, & Lord, 1995) in the *R. granulosa* species complex, in which closely related species tend to occupy more similar climatic niches than expected by random evolution (Desdevises et al., 2003; Losos, 2008), having also a more similar pattern of skull trait covariances. Given that differences in species P‐matrices are associated to differences in an external agent (climate), we argue that these differences are being caused by divergent patterns of external selection the species are subjected to. The climate‐related selection might be stabilizing selection acting directly on skull trait covariances or directional selection acting on the phenotypic means but having indirect effects on trait covariances. Simulations have shown that directional selection can change integration/modularity patterns (Melo & Marroig, 2015), and we have previously shown that directional selection acted on the phenotypic means of the toad species skulls (Simon, Machado & Marroig, 2016). Thus, the most basal species in the phylogeny, *R. fernandezae* and *R. dorbignyi*, potentially have a very similar pattern of external selection acting on skull trait covariance structure that diverges from the pattern of selection present in the more derived species, such as *R. merianae*,* R. humboldti,* and *R. centralis*. This pattern was detected independent of the analysis used to compare trait covariance structure among species (Random Skewers—RS or Relative Eigenanalysis—RE, see Figure 3b and Fig. S3), reinforcing that climatic factors that correlate with phylogeny probably are relevant to create divergence in covariation patterns and in the response of species to multivariate selection.

However, when exploring the effects of climate and phylogeny on species differences in trait covariance structure without isometric size variation, the phylogenetically structured climatic variation ceases to be relevant, but only when comparing species P‐matrices with RS. This result indicates that the phylogenetic component of climatic variation associated to P‐matrix dissimilarity is related to species differences in the amount of isometric size variation. Species that have high amounts of size variation will have higher similarity in their response to selection because the response vectors are biased toward directions that accumulate most variance (corresponding to Schluter's lines of least evolutionary resistance; Schluter, 1996; Marroig & Cheverud, 2005; Simon, Machado & Marroig, 2016). Therefore, species with more size variation will have a higher proportion of their response vectors aligned with size and consequently a higher similarity index. Yet, given that climatic variation independent of phylogeny explained part of the differences in species P‐matrices without isometric size variation (Table 4), there were probably independent responses of the species to external selection associated to climatic differences. The highest difference that we found between species exposed to divergent climates is related to the occipital and parasphenoid bones that compose the neurocranium functional unit. Species exposed to less temperature seasonality and higher mean temperatures and mean precipitation (positive coefficients in climatic PC1, see Figure 5b,c) have positive allometric growth of the occipital and parasphenoid bones (except *R. merianae*), while species exposed to the opposite climatic conditions have negative allometric growth (see Table S21). The occipital and parasphenoid bones are also bones with high sensitivity to T3 hormone (Hanken & Hall, 1988a), and T3 is known to influence growth in anurans (e.g.*,* Hayes, 1995). In addition, brain development is an early response to thyroxin hormone (Cai & Brown, 2003), as is the ossification of occipital and parasphenoid bones (Trueb & Hanken, 1992) that protect the ventral part of the skull. Hence, it is plausible that the divergent pattern of selection is related to divergence in allometric growth of the neurocranial unit by changes of the interaction between T3 regulation and growth hormones associated to different climates. However, other ecological factors besides climate are probably relevant to determine the pattern of selection, as most variation in species skull trait covariance was left unexplained in our analysis. Also, performing modularity analysis in a higher diversity of anurans will be important to generalize or not the patterns found in this study. It would be especially interesting to investigate modularity in anuran species that differ a great extension in the percentage of variation due to growth.

In conclusion, the process of growth instead of the extreme remodeling process that occurs during anuran metamorphosis is the main event that obscures developmental modularity due to embryonic origin of skull bones. Functional modularity seems relevant for the toad species, indicating that functional interactions among skull bones are subjected to selection and do shape trait correlations. Yet, given that hormonal‐related modules were detected in the toad species and that allometric growth and T3 hormonal regulation interact, hormonal regulation of metamorphosis also seems to mold some trait interactions at the phenotypic level. Environmental agents, such as climate, may also impose a correlation pattern on adult phenotypes by determining differences across species in the pattern of external stabilizing selection or directional selection. Differences in how much functional modularity is expressed in each species potentially interfere on how species respond to external selection, given that stabilizing selection shapes the phenotypic variation available for the action of external selective agents (Cheverud, 1984; Pélabon et al., 2011).

## CONFLICT OF INTEREST

None declared.

## AUTHOR'S CONTRIBUTIONS

MNS and GM conceived the ideas and designed the methodology; MNS collected the data; MNS and GM analyzed the data; MNS led the writing of the manuscript; MNS and GM gave the final approval for publication.

## DATA ACCESSIBILITY

The data supporting this work are deposited in Dryad: https://doi.org/10.5061/dryad.bs41q.

## References

[ece33592-bib-0002] Adams, D. C. (2014). A generalized K statistic for estimating phylogenetic signal from shape and other high‐dimensional multivariate data. Systematic Biology, 63, 685–697. https://doi.org/10.1093/sysbio/syu030 24789073 10.1093/sysbio/syu030

[ece33592-bib-0003] Armbruster, W. S. , Pelabon, C. , Bolstad, G. H. , & Hansen, T. F. (2014). Integrated phenotypes: Understanding trait covariation in plants and animals. Philosophical Transactions of the Royal Society B: Biological Sciences, 369, 20130245 https://doi.org/10.1098/rstb.2013.0245 10.1098/rstb.2013.0245 PMC4084533 25002693

[ece33592-bib-0004] Arnold, S. J. (1983). Morphology, performance and fitness. American Zoologist, 23, 347–361. https://doi.org/10.1093/icb/23.2.347

[ece33592-bib-0005] Arnold, S. J. , & Phillips, P. C. (1999). Hierarchical comparison of genetic variance‐covariance matrices. II. Coastal‐inland divergence in the garter snake, *Tamnophis elegans* . Evolution, 53, 1516–1527. https://doi.org/10.1111/j.1558-5646.1999.tb05415.x 28565546 10.1111/j.1558-5646.1999.tb05415.x

[ece33592-bib-0501] Arnold, S. J. (2005). The ultimate causes of phenotypic integration: Lost in translation. Evolution, 59, 2059–2061. https://doi.org/10.1554/BR05-8.1

[ece33592-bib-0006] Berg, R. L. (1960). The ecological significance of correlation pleiades. Evolution, 14, 171–180. https://doi.org/10.1111/evo.1960.14.issue-2

[ece33592-bib-0007] Blomberg, S. P. , Garland, T. Jr , & Ives, A. R. (2003). Testing for phylogenetic signal in comparative data: Behavioral traits are more labile. Evolution, 57, 717–745. https://doi.org/10.1111/evo.2003.57.issue-4 12778543 10.1111/j.0014-3820.2003.tb00285.x

[ece33592-bib-0008] Busby, J. R. (1991). BIOCLIM – A bioclimatic analysis and prediction system In Margules C. R., & Austin M. P. (Eds.), Nature conservation: Cost effective biological survey and data analysis (pp. 64–68). Melbourne, Vic: Csiro Publishing.

[ece33592-bib-0009] Cai, L. , & Brown, D. D. (2003). Expression of type II iodothyronine deiodinase marks the time that a tissue responds to thyroid hormone‐induced metamorphosis in *Xenopus laevis* . Developmental Biology, 226, 87–95. https://doi.org/10.1016/j.ydbio.2003.10.005 10.1016/j.ydbio.2003.10.005 14729480

[ece33592-bib-0010] Calsbeek, B. , & Goodnight, C. J. (2009). Empirical comparison of G matrix test statistics: Finding biologically relevant change. Evolution, 63, 2627–2635. https://doi.org/10.1111/evo.2009.63.issue-10 19490079 10.1111/j.1558-5646.2009.00735.x

[ece33592-bib-0011] Cheverud, J. M. (1982). Phenotypic, genetic, and environmental morphological integration in the cranium. Evolution, 36, 499 https://doi.org/10.1111/evo.1982.36.issue-3 28568050 10.1111/j.1558-5646.1982.tb05070.x

[ece33592-bib-0012] Cheverud, J. M. (1984). Quantitative genetics and developmental constraints on evolution by selection. Journal of Theoretical Biology, 110, 155–171. https://doi.org/10.1016/S0022-5193(84)80050-8 6492829 10.1016/s0022-5193(84)80050-8

[ece33592-bib-0014] Cheverud, J. M. (1996). Developmental integration and the evolution of pleiotropy. American Zoologist, 36, 44–50. https://doi.org/10.1093/icb/36.1.44

[ece33592-bib-0015] Cheverud, J. M. , & Marroig, G. (2007). Comparing covariance matrices: Random skewers method compared to the common principal components model. Genetics and Molecular Biology, 30, 461–469. https://doi.org/10.1590/S1415-47572007000300027

[ece33592-bib-0016] Cheverud, J. M. , Wagner, G. P. , & Dow, M. M. (1989). Methods for the comparative analysis of variation patterns. Systematic Zoology, 38, 201 https://doi.org/10.2307/2992282

[ece33592-bib-0017] Desdevises, Y. , Legendre, P. , Azouzi, L. , & Morand, S. (2003). Quantifying phylogenetically structured environmental variation. Evolution, 57, 2647–2652. https://doi.org/10.1111/evo.2003.57.issue-11 14686540 10.1111/j.0014-3820.2003.tb01508.x

[ece33592-bib-0018] Diniz‐Filho, J. A. F. , de Sant'Ana, C. E. R. , & Bini, L. M. (1998). An eigenvector method for estimating phylogenetic inertia. Evolution, 52, 1247–1262. https://doi.org/10.1111/evo.1998.52.issue-5 28565378 10.1111/j.1558-5646.1998.tb02006.x

[ece33592-bib-0019] Duran, A. , & Pie, M. R. (2015). Tempo and mode of climatic niche evolution in Primates. Evolution, 69, 1247–1262. https://doi.org/10.1111/evo.12730 10.1111/evo.12730 26178157

[ece33592-bib-0020] Emerson, B. E. (1982). Frog postcranial morphology: Identification of a functional complex. Copeia, 1982, 603–613. https://doi.org/10.2307/1444660

[ece33592-bib-0021] Falconer, D. S. , & Mackay, T. F. C. (1996). Introduction to quantitative genetics (4th ed.). Essex, UK: Longman Group Ltd p. 464.

[ece33592-bib-0022] Gallardo, J. M. (1965). The species *Bufo granulosus* Spix (Salientia: Bufonidae) and its geographic variation. Bulletin of the Museum of Comparative Zoology, 134, 107–138. https://doi.org/10.5962/bhl.part.20069

[ece33592-bib-0023] Gaudin, A. J. (1978). The sequence of cranial ossification in the California Toad, *Bufo boreas* (Amphibia, Anura, Bufonidae). Journal of Herpetology, 12, 309–318. https://doi.org/10.2307/1563611

[ece33592-bib-0024] Genz, A. , Bretz, F. , Hothorn, T. , Miwa, T. , Mi, X. , Leisch, F. , & Scheipl, F. (2008). mvtnorm: Multivariate normal and T distribution. URL hppt://CRAN.R-project.org. R package version 0.9‐0.

[ece33592-bib-0025] Goswami, A. , & Finarelli, J. A. (2016). EMMLi: A maximum likelihood approach to the analysis of modularity. Evolution, 70, 1622–1637. https://doi.org/10.1111/evo.12956 27188434 10.1111/evo.12956

[ece33592-bib-0026] Grafen, A. (1989). The phylogenetic regression. Philosophical Transactions of the Royal Society B: Biological Sciences, 326, 119–127. https://doi.org/10.1098/rstb.1989.0106 10.1098/rstb.1989.0106 2575770

[ece33592-bib-0027] Haas, A. (2001). Mandibular arch musculature of anuran tadpoles, with comments on homologies of amphibian jaw muscles. Journal of Morphology, 247, 1–33. https://doi.org/10.1002/(ISSN)1097-4687 11124683 10.1002/1097-4687(200101)247:1<1::AID-JMOR1000>3.0.CO;2-3

[ece33592-bib-0028] Hallgrímsson, B. , Jamniczky, H. , Young, N. M. , Rolian, C. , Parsons, T. E. , Boughner, J. C. , & Marcucio, R. S. (2009). Deciphering the palimpsest: Studying the relationship between morphological integration and phenotypic covariation. Evolutionary Biology, 36, 355–376. https://doi.org/10.1007/s11692-009-9076-5 23293400 10.1007/s11692-009-9076-5 PMC3537827

[ece33592-bib-0029] Halpern, M. , & Martinez‐Marcos, A. (2003). Structure and function of the vomeronasal system: An update. Progress in Neurobiology, 70, 245–318. https://doi.org/10.1016/S0301-0082(03)00103-5 12951145 10.1016/s0301-0082(03)00103-5

[ece33592-bib-0030] Hanken, J. , & Hall, B. K. (1988a). Skull development during anuran metamorphosis: II. Role of thyroid hormone in osteogenesis. Anatomy and Embryology, 178, 219–227. https://doi.org/10.1007/BF00318225 3414976 10.1007/BF00318225

[ece33592-bib-0031] Hanken, J. , & Hall, B. K. (1988b). Skull development during anuran metamorphosis: I. Early development of the first three bones to form – The exoccipital, the parasphenoid, and the frontoparietal. Journal of Morphology, 195, 247–256. https://doi.org/10.1002/(ISSN)1097-4687 3379643 10.1002/jmor.1051950303

[ece33592-bib-0032] Hanken, J. , & Summers, C. H. (1988). Skull development during anuran metamorphosis: III. Role of thyroid hormone in chondrogenesis. Journal of Experimental Zoology, 246, 156–170. https://doi.org/10.1002/(ISSN)1097-010X 3392514 10.1002/jez.1402460208

[ece33592-bib-0033] Hansen, T. , & Houle, D. (2004). Evolvability, stabilizing selection, and the problem of stasis In Preston K., & Pigliucci M. (Eds.), Phenotypic integration: Studying the ecology and evolution of complex phenotypes (pp. 130–150). Oxford, UK: Oxford University Press.

[ece33592-bib-0034] Hayes, T. B. (1995). Interdependence of corticosterone and thyroid hormones in larval toads (*Bufo boreas*). I. Thyroid hormone‐dependent and independent effects of corticosterone on growth and development. The Journal of Experimental Zoology, 271, 95–102. https://doi.org/10.1002/(ISSN)1097-010X 7884391 10.1002/jez.1402710204

[ece33592-bib-0035] Hijmans, R. J. , Cameron, S. E. , Parra, J. L. , Jones, P. G. , & Jarvis, A. (2005). Very high resolution interpolated climate surfaces for global land areas. International Journal of Climatology, 25, 1965–1978. https://doi.org/10.1002/(ISSN)1097-0088

[ece33592-bib-0036] Jared, C. , Antoniazzi, M. M. , Navas, C. A. , Katchburian, E. , Freymüller, E. , Tambourgi, D. V. , & Rodrigues, M. T. (2005). Head co‐ossification, phragmosis and defence in the casque‐headed tree frog *Corythomantis greeningi* . Journal of Zoology, 265, 1–8. https://doi.org/10.1017/S0952836904005953

[ece33592-bib-0037] Jolicoeur, P. (1963). Note: The multivariate generalization of the allometry equation. Biometrics, 19, 497–499. https://doi.org/10.2307/2527939

[ece33592-bib-0038] Jungblut, L. D. , Pozzi, A. G. , & Paz, D. A. (2011). Larval development and metamorphosis of the olfactory and vomeronasal organs in the toad *Rhinella (Bufo)arenarum* (Hensel, 1867). Acta Zoologica, 92, 305–315. https://doi.org/10.1111/azo.2011.92.issue-4

[ece33592-bib-0039] Kathe, W. (1999). Comparative morphology and functional interpretation of the sutures in the dermal skull roof of temnospondyl amphibians. Zoological Journal of the Linnean Society, 126, 1–39. https://doi.org/10.1111/zoj.1999.126.issue-1

[ece33592-bib-0040] Kerney, R. R. , Brittain, A. L. , Hall, B. K. , & Buchholz, D. R. (2012). Cartilage on the move: Cartilage lineage tracing during tadpole metamorphosis. Development, Growth & Differentiation, 54, 739–752. https://doi.org/10.1111/dgd.2012.54.issue-8 10.1111/dgd.12002 PMC3488034 23036161

[ece33592-bib-0041] Klingenberg, C. P. (2008). Morphological integration and developmental modularity. Annual Review of Ecology, Evolution, and Systematics, 39, 115–132. https://doi.org/10.1146/annurev.ecolsys.37.091305.110054

[ece33592-bib-0042] Klingenberg, C. P. , Debat, V. , & Roff, D. A. (2010). Quantitative genetics of shape in cricket wings: Developmental integration in a functional structure. Evolution, 64, 2935–2951. https://doi.org/10.1111/j.1558-5646.2010.01030.x 20482613 10.1111/j.1558-5646.2010.01030.x

[ece33592-bib-0043] Lande, R. (1979). Quantitative genetic analysis of multivariate evolution, applied to brain: Body size allometry. Evolution, 33, 402–412. https://doi.org/10.2307/2407630 28568194 10.1111/j.1558-5646.1979.tb04694.x

[ece33592-bib-0044] Lande, R. (1980). The genetic covariance between characters maintained by pleiotropic mutations. Genetics, 94, 203–215. 17248993 10.1093/genetics/94.1.203 PMC1214134

[ece33592-bib-0046] Legendre, P. , & Legendre, L. (1998). Numerical ecology (2nd ed., p. 853). Amsterdam, Netherlands: Elsevier Science B. V..

[ece33592-bib-0047] Lessells, C. M. , & Boag, P. T. (1987). Unrepeatable repeatabilities: A common mistake. The Auk, 104, 116–121. https://doi.org/10.2307/4087240

[ece33592-bib-0048] Losos, J. B. (1990). The evolution of form and function: Morphology and locomotor performance in West Indian *Anolis* lizards. Evolution, 44, 1189 https://doi.org/10.1111/evo.1990.44.issue-5 28563896 10.1111/j.1558-5646.1990.tb05225.x

[ece33592-bib-0049] Losos, J. B. (2008). Phylogenetic niche conservatism, phylogenetic signal and the relationship between phylogenetic relatedness and ecological similarity among species. Ecology Letters, 11, 995–1003. https://doi.org/10.1111/ele.2008.11.issue-10 18673385 10.1111/j.1461-0248.2008.01229.x

[ece33592-bib-0050] Manly, B. F. J. (2004). Randomization, bootstrap and Monte Carlo methods in biology (2nd ed., pp. 480). Boca Raton, FL: Chapman & Hall/CRC.

[ece33592-bib-0051] Márquez, E. J. (2008). A statistical framework for testing modularity in multidimensional data. Evolution, 62, 2688–2708. https://doi.org/10.1111/evo.2008.62.issue-10 18691262 10.1111/j.1558-5646.2008.00476.x

[ece33592-bib-0052] Márquez, E. J. , Cabeen, R. , Woods, R. P. , & Houle, D. (2012). The measurement of local variation in shape. Evolutionary Biology, 39, 419–439. https://doi.org/10.1007/s11692-012-9159-6 23180896 10.1007/s11692-012-9159-6 PMC3501737

[ece33592-bib-0053] Marroig, G. , & Cheverud, J. M. (2005). Size as a line of least evolutionary resistance: Diet and adaptive morphological radiation in New World monkeys. Evolution, 59, 1128–1142. https://doi.org/10.1111/evo.2005.59.issue-5 16136810

[ece33592-bib-0054] Marroig, G. , Melo, D. A. R. , & Garcia, G. (2012). Modularity, noise, and natural selection. Evolution, 66, 1506–1524. https://doi.org/10.1111/j.1558-5646.2011.01555.x 22519787 10.1111/j.1558-5646.2011.01555.x

[ece33592-bib-0055] Marroig, G. , Vivo, M. , & Cheverud, J. M. (2004). Cranial evolution in sakis (*Pithecia*, Platyrrhini) II: Evolutionary processes and morphological integration: Cranial evolution in sakis. Journal of Evolutionary Biology, 17, 144–155. https://doi.org/10.1046/j.1420-9101.2003.00653.x 15000657 10.1046/j.1420-9101.2003.00653.x

[ece33592-bib-0056] Melo, D. , & Marroig, G. (2015). Directional selection can drive the evolution of modularity in complex traits. Proceedings of the National Academy of Science of the USA, 13, 470–475. https://doi.org/10.1073/pnas.1322632112 10.1073/pnas.1322632112 PMC4299217 25548154

[ece33592-bib-0502] Melo, D. , Garcia, G. , Hubbe, A. , Assis, A. P. , & Marroig, G. (2015). EvolQG – An R package for evolutionary quantitative genetics. F1000 Research, 4, 925 https://doi.org/10.12688/f1000research.7082.2 27785352 10.12688/f1000research.7082.1 PMC5022708

[ece33592-bib-0057] Mitteroecker, P. , & Bookstein, F. (2007). The conceptual and statistical relationship between modularity and morphological integration. Systematic Biology, 56, 818–836. https://doi.org/10.1080/10635150701648029 17934997 10.1080/10635150701648029

[ece33592-bib-0058] Mitteroecker, P. , & Bookstein, F. (2009). The ontogenetic trajectory of the phenotypic covariance matrix, with examples from craniofacial shape in rats and humans. Evolution, 63, 727–737. https://doi.org/10.1111/evo.2009.63.issue-3 19087182 10.1111/j.1558-5646.2008.00587.x

[ece33592-bib-0059] Monteiro, L. R. , Bonato, V. , & Dos Reis, S. F. (2005). Evolutionary integration and morphological diversification in complex morphological structures: Mandible shape divergence in spiny rats (Rodentia, Echimyidae). Evolution & Development, 7, 429–439. https://doi.org/10.1111/ede.2005.7.issue-5 16174036 10.1111/j.1525-142X.2005.05047.x

[ece33592-bib-0060] Monteiro, L. R. , & Nogueira, M. R. (2010). Adaptive radiations, ecological specialization, and the evolutionary integration of complex morphological structures. Evolution, 64, 724–744. https://doi.org/10.1111/evo.2010.64.issue-3 19804403 10.1111/j.1558-5646.2009.00857.x

[ece33592-bib-0061] Narvaes, P. (2003). Revisão taxonômica das espécies de Bufo do complexo granulosa. Ph.D. dissertation.

[ece33592-bib-0062] Narvaes, P. , & Rodrigues, M. T. (2009). Taxonomic revision of *Rhinella granulosa* species group (Amphibia, Anura, Bufonidae), with a description of a new species. Arquivos de Zoologia, 40, 1–73. https://doi.org/10.11606/issn.2176-7793.v40i1p1-73

[ece33592-bib-0063] Navas, C. A. , Jared, C. , & Antoniazzi, M. M. (2002). Water economy in the casque‐headed tree‐frog *Corythomantis greeningi* (Hylidae): Role of behaviour, skin, and skull skin co‐ossification. Journal of Zoology, 257, 525–532. https://doi.org/10.1017/S0952836902001103

[ece33592-bib-0064] Nishikawa, K. C. (1999). Neuromuscular control of prey capture in frogs. Philosophical Transactions of the Royal Society of London B: Biological Sciences, 354, 941–954. https://doi.org/10.1098/rstb.1999.0445 10382226 10.1098/rstb.1999.0445 PMC1692590

[ece33592-bib-0065] Nishikawa, K. C. , & Gans, C. (1996). Mechanisms of tongue protraction and narial closure in the marine toad *Bufo marinus* . Journal of Experimental Biology, 199, 2511–2529. 9114504 10.1242/jeb.199.11.2511

[ece33592-bib-0503] Oksanen, J. , Kindt, R. , Legendre, P. , O'Hara, B. , Henry, M. , & Stevens, H. (2007). ‘vegan’: community ecology package. URL http://cran.r-project.org/, http://r-forge.r-project.org/projects/vegan/

[ece33592-bib-0066] Olson, E. C. , & Miller, R. L. (1958). Morphological integration (1st ed., p. 376). Chicago, IL: The University of Chicago Press.

[ece33592-bib-0067] Pélabon, C. , Armbruster, W. S. , & Hansen, T. F. (2011). Experimental evidence for the Berg hypothesis: Vegetative traits are more sensitive than pollination traits to environmental variation. Functional Ecology, 25, 247–257. https://doi.org/10.1111/j.1365-2435.2010.01770.x

[ece33592-bib-0068] Pereyra, M. O. , Baldo, D. , Blotto, B. L. , Iglesias, P. P. , Thomé, M. T. C. , Haddad, C. F. B. , … Faivovich, J. (2015). Phylogenetic relationships of toads of the *Rhinella granulosa* group (Anura: Bufonidae): A molecular perspective with comments on hybridization and introgression. Cladistics, 32, 36–53. https://doi.org/10.1111/cla.12110 10.1111/cla.12110 34732018

[ece33592-bib-0069] Piekarski, N. , Gross, J. B. , & Hanken, J. (2014). Evolutionary innovation and conservation in the embryonic derivation of the vertebrate skull. Nature Communications, 5, 5661 https://doi.org/10.1038/ncomms6661 10.1038/ncomms6661 PMC4251486 25434971

[ece33592-bib-0070] Pigliucci, M. , & Preston, K. (2004). Phenotypic integration: Studying the ecology and evolution of complex phenotypes (p. 464). Oxford, UK: Oxford University Press.

[ece33592-bib-0071] Porto, A. , de Oliveira, F. B. , Shirai, L. T. , De Conto, V. , & Marroig, G. (2009). The evolution of modularity in the mammalian skull I: Morphological integration patterns and magnitudes. Evolutionary Biology, 36, 118–135. https://doi.org/10.1007/s11692-008-9038-3

[ece33592-bib-0504] Porto, A. , Shirai, L. T. , Oliveira, F. B. , & Marroig, G. (2013). Size variation, growth strategies, and the evolution of modularity in the mammalian skull. Evolution, 67, 3305–3322. https://doi.org/10.111/evo.12177 24152009 10.1111/evo.12177

[ece33592-bib-0072] Revell, L. J. (2012). phytools: An R package for phylogenetic comparative biology (and other things). Methods in Ecology and Evolution, 3, 217–223. https://doi.org/10.1111/j.2041-210X.2011.00169.x

[ece33592-bib-0073] Riedl, R. (1977). A systems‐analytical approach to macro‐evolutionary phenomena. The Quarterly Review of Biology, 52, 351–370. https://doi.org/10.1086/410123 343152 10.1086/410123

[ece33592-bib-0074] Riedl, R. (1978). Order in living organisms: Systems analysis of evolution (p. 334). New York, NY: John Wiley and Sons Ltd.

[ece33592-bib-0075] Rose, C. S. , & Reiss, J. O. (1993). Metamorphosis and the vertebrate Skull: Ontogenetic pattern and developmental mechanisms In Hanken J., & Hall B. K. (Eds.), The skull volume 1: Development (pp. 289–346). Chicago, IL: The University of Chicago Press.

[ece33592-bib-0076] Sanuy, D. , & Joly, P. (2009). Olfactory cues and breeding habitat selection in the natterjack toad, *Bufo calamita* . Amphibia‐Reptilia, 30, 555–559. https://doi.org/10.1163/156853809789647158

[ece33592-bib-0077] Schluter, D. (1996). Adaptive radiation along genetic lines of least resistance. Evolution, 50, 1766–1774. https://doi.org/10.1111/evo.1996.50.issue-5 28565589 10.1111/j.1558-5646.1996.tb03563.x

[ece33592-bib-0078] Schunke, A. C. , Bromiley, P. A. , Tautz, D. , & Thacker, N. A. (2012). TINA manual landmarking tool: Software for the precise digitization of 3D landmarks. Frontiers in Zoology, 9, 6 https://doi.org/10.1186/1742-9994-9-6 22480150 10.1186/1742-9994-9-6 PMC3353871

[ece33592-bib-0079] Schwenk, K. , & Wagner, G. P. (2001). Function and the evolution of phenotypic stability: Connecting pattern to process. American Zoologist, 41, 552–563. https://doi.org/10.1093/icb/41.3.552

[ece33592-bib-0080] Seibert, E. A. , Lillywhite, H. B. , & Wassersug, R. J. (1974). Cranial coossification in frogs: Relationship to rate of evaporative water loss. Physiological Zoology, 47, 261–265. https://doi.org/10.1086/physzool.47.4.30152529

[ece33592-bib-0081] Simon, M. N. , Machado, F. A. , & Marroig, G. (2016). High evolutionary constraints limited adaptive responses to past climate changes in toad skulls. Proceedings of the Royal Society B: Biological Sciences, 283, 20161783 https://doi.org/10.1098/rspb.2016.1783 27798306 10.1098/rspb.2016.1783 PMC5095385

[ece33592-bib-0082] Simon, M. N. , & Marroig, G. (2015). Landmark precision and reliability and accuracy of linear distances estimated using 3D computed micro‐tomography and the open‐source TINA Manual Landmarking Tool software. Frontiers in Zoology, 12, 12 https://doi.org/10.1186/s12983-015-0101-5 26120349 10.1186/s12983-015-0101-5 PMC4481120

[ece33592-bib-0083] Somers, K. M. (1989). Allometry, isometry and shape in principal components analysis. Systematic Zoology, 38, 169–173. https://doi.org/10.2307/2992386

[ece33592-bib-0084] Thomson, K. S. (1993). Segmentation, the adult skull and the problem of homology In Hanken J., & Hall B. K. (Eds.), The skull volume 2: Patterns of structural and systematic diversity (pp. 36–68). Chicago, IL: The University of Chicago Press.

[ece33592-bib-0085] Trueb, L. (1993). Patterns of cranial diversity among the Lissamphibia In Hanken J., & Hall B. K. (Eds.), The skull volume 2: Patterns of structural and systematic diversity (pp. 255–343). Chicago, IL: The University of Chicago Press.

[ece33592-bib-0086] Trueb, L. , & Hanken, J. (1992). Skeletal development in *Xenopus laevis* (Anura: Pipidae). Journal of Morphology, 214, 1–41. https://doi.org/10.1002/(ISSN)1097-4687 1433306 10.1002/jmor.1052140102

[ece33592-bib-0087] van der Linde, K. , & Houle, D. (2009). Inferring the nature of allometry from geometric data. Evolutionary Biology, 36, 311–322. https://doi.org/10.1007/s11692-009-9061-z

[ece33592-bib-0088] Waddington, C. H. (1957). The strategy of the genes: A discussion of some aspects of theoretical biology (p. 262). Abingdon, UK: Routledge.

[ece33592-bib-0089] Wagner, G. P. (1996). Homologues, natural kinds and the evolution of modularity. American Zoologist, 36, 36–43. https://doi.org/10.1093/icb/36.1.36

[ece33592-bib-0090] Wagner, G. P. , & Altenberg, L. (1996). Perspective: Complex adaptations and the evolution of evolvability. Evolution, 50, 967 https://doi.org/10.1111/evo.1996.50.issue-3 28565291 10.1111/j.1558-5646.1996.tb02339.x

[ece33592-bib-0091] Wagner, G. P. , Pavlicev, M. , & Cheverud, J. M. (2007). The road to modularity. Nature Reviews Genetics, 8, 921–931. https://doi.org/10.1038/nrg2267 10.1038/nrg2267 18007649

[ece33592-bib-0092] Wainwright, P. C. (2007). Functional versus morphological diversity in macroevolution. Annual Review of Ecology, Evolution, and Systematics, 38, 381–401. https://doi.org/10.1146/annurev.ecolsys.38.091206.095706

[ece33592-bib-0093] West‐Eberhard, M. J. (2003). Developmental plasticity and evolution (1st ed., p. 794). Oxford, UK: Oxford University Press.

[ece33592-bib-0094] Westoby, M. , Leishman, M. R. , & Lord, J. M. (1995). On misinterpreting the ‘phylogenetic correction’. The Journal of Ecology, 83, 531 https://doi.org/10.2307/2261605

[ece33592-bib-0095] Young, R. L. , & Badyaev, A. V. (2006). Evolutionary persistence of phenotypic integration: Influence of developmental and functional relationships on complex trait evolution. Evolution, 60, 1291–1299. https://doi.org/10.1111/evo.2006.60.issue-6 16892978

[ece33592-bib-0505] Young, N. , & Hallgrimsson, B. (2005). Serial homology and the evolution of mammalian limb covariation structure. Evolution, 59, 2691–2704. https://doi.org/10.1554/05-233.1 16526515

[ece33592-bib-0096] Zelditch, M. L. , & Swiderski, D. L. (2011). Epigenetic interactions: The developmental route to functional integration In Hallgrímsson B., & Hall B. K. (Eds.), Epigenetics: Linking genotype and phenotype in development and evolution (pp. 290–316). Berkeley, CA: University of California Press.

[ece33592-bib-0097] Zelditch, M. L. , Swiderski, D. L. , & Sheets, H. D. (1998). Geometric morphometrics for biologists: A primer (2nd ed., p. 478). London, UK: Academic Press.

[ece33592-bib-0098] Zelditch, M. L. , Wood, A. R. , Bonett, R. M. , & Swiderski, D. L. (2008). Modularity of the rodent mandible: Integrating bones, muscles, and teeth. Evolution & Development, 10, 756–768. https://doi.org/10.1111/j.1525-142X.2008.00290.x 19021747 10.1111/j.1525-142X.2008.00290.x

