# Supplementary material for: Evolution of a complex phenotype with biphasic ontogeny: Contribution of development versus function and climatic variation to skull modularity in toads
Source: Ecol Evol. 2017 Nov 7;7(24):10752–69. doi: 10.1002/ece3.3592 (PMC5743631; doi:10.1002/ece3.3592)
Supplement: Supplementary file 1 [file ECE3-7-10752-s001.pdf]

Supplementary Information

Supplementary methods

1. Removal of isometric size variation

To remove isometric size variation, we first log-transformed the raw linear distances and we subtracted the lines' and columns' means from each distance (Somers 1989). Then, we extracted the residuals from the linear models controlling for sex and geography. Because the residuals are centered in zero, we added the mean distance values to each individual distance so we could later subtract from them the corresponding individual geometric mean. After all these steps, we constructed new species P-matrices that have their last eigenvector as an isometric vector (all coefficients have the same value) with zero eigenvalue (i.e., no isometric variation).

Supplementary Tables

Table S1. Sample sizes and sources of variation in the skull distances' means that were removed before constructing species P-matrices. All species belong to the *R. granulosa* complex except *R. margaritifera*. **S** is for sexual dimorphism and **G** for geographical variation within species. The species P-matrices were constructed with the residuals extracted from linear models controlling for these sources of variation.

Species	Sample size	Source of variation
R. bergi	45	G
R. centralis	38	G
R. dorbignyi	100	S, G
R. fernandezae	125	S x G
R. granulosa	189	S, G
R. humboldti	71	S x G
R. major	205	S, G
R. margaritifera	38	S
R. merianae	70	S, G
R. mirandaribeiroi	125	S x G
R. pygmaea	58	G

Table S2. Description of the 3D landmarks placed on the toad skulls. Every specimen was scanned with a micro-CT system to construct 3D volumes of their skulls. Landmarks are in bone sutures or tip of bones and spread through the whole skull. Landmarking procedure was done twice in each specimen with the use of TINA manual landmarking software.

Landmarks	Description	Position	View
1	Anterior tip of nasal bone	midline	dorsal
2	Nasal and frontoparietal suture	midline	dorsal
3	Posterior tip of frontoparietal suture	midline	dorsal
4	Nasal and maxillary suture	right, left	dorsal
5	Nasal and frontoparietal lateral suture	right, left	dorsal
6	Frontoparietal and squamosal suture	right, left	dorsal
7	Frontoparietal, squamosal and occipital suture	right, left	dorsal
8	Squamosal and occipital suture	right, left	dorsal
9	Frontoparietal and occipital suture	right, left	dorsal
10	Prenasal and maxillary suture	right, left	lateral
11	Nasal and maxillary lateral suture	right, left	lateral
12	Squamosal and maxillary suture	right, left	lateral
13	Sphenethmoid and parasphenoid suture	midline	ventral
14	Posterior tip of parasphenoid corpus	midline	ventral
15	Anterior tip of premaxillary bone	right, left	ventral
16	Premaxillary and maxillary suture	right, left	ventral
17	Pterygoid and maxillary suture	right, left	ventral
18	Neopalatine and sphenethmoid suture	right, left	ventral
19	Tip of pterygoid process	right, left	ventral
20	Pterygoid and parasphenoid suture	right, left	ventral
21	Anterior tip of mandibular bone	right, left	ventral
22	Posterior tip of mandibular bone	right, left	ventral

Table S3. Phylogenetic distances among the toad species of the *R. granulosa* complex. Cophenetic distances were calculated from a Bayesian molecular phylogeny (Pereyra et al. 2014). Distances are the sum of the branch lengths between a pair of tip species and their most recent common ancestor.

	1	2	3	4	5	6	7	8	9	10
**1. *R. bergi***										
**2. *R. centralis***	0.07									
**3. *R. dorbignyi***	0.05	0.08								
**4. *R. fernandezae***	0.05	0.08	0.00							
**5. *R. granulosa***	0.06	0.07	0.07	0.07						
**6. *R. humboldti***	0.07	0.05	0.07	0.07	0.06					
**7. *R. major***	0.05	0.07	0.06	0.05	0.06	0.06				
**8. *R. merianae***	0.06	0.05	0.06	0.06	0.06	0.04	0.05			
**9. *R. mirandaribeiroi***	0.07	0.08	0.07	0.07	0.05	0.07	0.06	0.06		
**10. *R. pygmaea***	0.05	0.08	0.05	0.05	0.07	0.07	0.06	0.07	0.08	

Table S4. First Climatic Principal Component (PC1). Climatic PC1 was extracted from a climatic correlation matrix constructed with species mean climatic variables (WorldClim). The variables were z-scored transformed to avoid biases due to their very different scales. Highest coefficients are in bold.

Climatic Variables	Description	Scale	PC1
BIO1	Annual Mean Temperature	oC * 10	**0.33**
BIO2	Mean Diurnal Range (Mean of monthly (max temp - min temp))	oC * 10	**-0.12**
BIO4	Temperature Seasonality (standard deviation)	oC * 10	**-0.30**
BIO5	Maximum Temperature of Warmest Month	oC * 10	**0.19**
BIO6	Minimum Temperature of Coldest Month	oC * 10	**0.32**
BIO8	Mean Temperature of Wettest Quarter	oC * 10	**0.25**
BIO9	Mean Temperature of Driest Quarter	oC * 10	**0.31**
BIO10	Mean Temperature of Warmest Quarter	oC * 10	**0.28**
BIO11	Mean Temperature of Coldest Quarter	oC * 10	**0.33**
BIO12	Annual Precipitation	mm	**0.20**
BIO13	Precipitation of Wettest Month	mm	**0.28**
BIO14	Precipitation of Driest Month	mm	-0.11
BIO15	Precipitation Seasonality (Coefficient of Variation)	mm	**0.23**
BIO16	Precipitation of Wettest Quarter	mm	**0.28**
BIO17	Precipitation of Driest Quarter	mm	-0.09
BIO18	Precipitation of Warmest Quarter	mm	-0.01
BIO19	Precipitation of Coldest Quarter	mm	**0.18**

Table S5. Percentage of allometric and isometric variation in each species.

Species	% allometric variation	% isometric variation
R. centralis	82	80
R. humboldti	71	71
R. merianae	64	60
R. granulosa	84	81
R. mirandaribeiroi	72	68
R. major	79	74
R. bergi	82	77
R. pygmaea	64	57
R. dorbignyi	78	70
R. fernandezae	80	72
R. margaritifera	51	36

Table S6. Empirical and lower and upper bound IC95 values for AVG diff for the developmental units tested with size variation. IC95 were constructed with 1,000 resampled P-matrices for each species. Significant AVG diff values are in bold (IC95 does not have zero).

DEVELOPMENTAL MODEL		AVG diff = [AVG +] - [AVG -]							
**with size**		**Branchial**	**Hyoid I**	**Hyoid II**	**Hyoid III**	**Mandibular I**	**Mandibular II**	**Mandibular III**	**Total II**
R. bergi	empirical	-0.055	-0.040	0.018	-0.018	-0.026	0.031	-0.001	0.029
	lower	-0.167	-0.079	-0.024	-0.062	-0.061	-0.013	-0.040	-0.010
	upper	0.036	-0.004	0.062	0.028	0.007	0.079	0.039	0.072
R. centralis	empirical	-0.144	-0.038	0.022	0.004	-0.066	-0.017	-0.024	-0.008
	lower	-0.278	-0.080	-0.021	-0.045	-0.103	-0.059	-0.067	-0.048
	upper	-0.044	-0.001	0.070	0.050	-0.034	0.023	0.017	0.032
R. dorbignyi	empirical	-0.264	-0.110	0.028	-0.160	0.003	**0.137**	-0.027	**0.090**
	lower	-0.379	-0.141	-0.011	-0.197	-0.029	0.106	-0.063	0.059
	upper	-0.161	-0.080	0.064	-0.120	0.038	0.167	0.012	0.120
R. fernandezae	empirical	-0.256	-0.070	**0.047**	-0.090	-0.029	**0.098**	-0.042	**0.078**
	lower	-0.351	-0.100	0.016	-0.127	-0.052	0.065	-0.070	0.053
	upper	-0.160	-0.038	0.076	-0.049	-0.002	0.127	-0.009	0.101
R. granulosa	empirical	-0.043	-0.024	0.010	0.004	-0.024	-0.003	0.008	0.002
	lower	-0.078	-0.038	-0.007	-0.015	-0.040	-0.020	-0.008	-0.012
	upper	-0.007	-0.010	0.028	0.022	-0.009	0.011	0.024	0.014
R. humboldti	empirical	-0.053	0.020	**0.059**	**0.047**	-0.062	-0.040	-0.039	0.001
	lower	-0.151	-0.022	0.014	0.004	-0.096	-0.081	-0.085	-0.031
	upper	0.040	0.062	0.105	0.091	-0.028	0.001	0.007	0.034
R. major	empirical	-0.124	-0.051	0.002	-0.022	-0.023	**0.021**	0.014	0.010
	lower	-0.182	-0.069	-0.019	-0.043	-0.041	0.001	-0.006	-0.007
	upper	-0.074	-0.034	0.025	0.000	-0.005	0.040	0.035	0.029
R. margaritifera	empirical	-0.088	-0.051	-0.004	-0.100	0.092	**0.143**	0.082	**0.085**
	lower	-0.277	-0.114	-0.091	-0.167	0.015	0.067	-0.009	0.024
	upper	0.121	0.016	0.086	-0.020	0.163	0.222	0.165	0.159
R. merianae	empirical	-0.039	-0.054	**0.061**	-0.023	-0.062	0.002	-0.003	0.035
	lower	-0.174	-0.094	0.003	-0.069	-0.106	-0.042	-0.057	-0.013
	upper	0.081	-0.011	0.127	0.026	-0.017	0.046	0.055	0.083
R. mirandaribeiroi	empirical	-0.020	-0.037	0.009	0.017	-0.026	0.007	0.022	0.013
	lower	-0.088	-0.065	-0.025	-0.013	-0.054	-0.023	-0.008	-0.015
	upper	0.044	-0.009	0.043	0.051	0.002	0.037	0.055	0.041
R. pygmaea	empirical	-0.116	-0.115	-0.044	-0.054	0.023	**0.084**	**0.090**	0.027
	lower	-0.266	-0.157	-0.103	-0.113	-0.034	0.023	0.034	-0.025
	upper	0.024	-0.067	0.018	0.015	0.082	0.146	0.153	0.076

Table S7. Empirical and IC95 values for AVG diff for the hormonal units tested with size variation. IC95 were constructed with 1,000 resampled P-matrices for each species. Significant AVG diff values are in bold (IC95 does not have zero).

**HORMONAL MODEL**		**AVG diff = [AVG +] - [AVG -]**			
**with size**		**T3 +++**	**T3 ++**	**T3 +**	**Total**
R. bergi	empirical	-0.067	0.033	**0.102**	0.028
	lower	-0.148	-0.009	0.053	0.000
	upper	0.000	0.084	0.157	0.062
R. centralis	empirical	-0.140	**0.074**	**0.055**	**0.042**
	lower	-0.231	0.036	0.004	0.017
	upper	-0.066	0.121	0.106	0.073
R. dorbignyi	empirical	-0.124	**0.079**	**0.140**	**0.060**
	lower	-0.192	0.041	0.105	0.034
	upper	-0.054	0.117	0.178	0.089
R. fernandezae	empirical	-0.127	**0.045**	**0.096**	**0.025**
	lower	-0.186	0.016	0.058	0.007
	upper	-0.069	0.072	0.133	0.043
R. granulosa	empirical	-0.046	**0.025**	**0.045**	**0.018**
	lower	-0.075	0.010	0.023	0.008
	upper	-0.019	0.041	0.067	0.028
R. humboldti	empirical	-0.018	**0.041**	0.011	**0.033**
	lower	-0.087	-0.004	-0.056	0.003
	upper	0.055	0.083	0.074	0.063
R. major	empirical	-0.093	**0.045**	**0.097**	**0.032**
	lower	-0.129	0.025	0.071	0.019
	upper	-0.061	0.064	0.123	0.046
R. margaritifera	empirical	-0.056	-0.030	**0.271**	-0.003
	lower	-0.192	-0.111	0.163	-0.056
	upper	0.079	0.054	0.374	0.056
R. merianae	empirical	-0.070	**0.089**	0.059	**0.070**
	lower	-0.160	0.036	-0.006	0.035
	upper	0.023	0.148	0.124	0.109
R. mirandaribeiroi	empirical	-0.058	**0.037**	**0.094**	**0.032**
	lower	-0.111	0.006	0.052	0.010
	upper	-0.008	0.070	0.135	0.055
R. pygmaea	empirical	-0.077	0.046	**0.181**	**0.047**
	lower	-0.175	-0.018	0.104	0.002
	upper	0.021	0.117	0.254	0.096

Table S8. Empirical and IC95 values for AVG diff for the functional units tested with size variation. IC95 were constructed with 1,000 resampled P-matrices for each species. Significant AVG diff values are in bold (IC95 does not have zero).

**FUNCTIONAL MODEL**		**AVG diff = [AVG +] - [AVG -]**							
**with size**		**Neurocranium**	**Orbit**	**Roof**	**Snout**	**Suspensorium I**	**Suspensorium II**	**Total I**	**Total II**
R. bergi	empirical	-0.067	-0.055	-0.019	**0.062**	0.042	-0.046	0.011	-0.008
	lower	-0.146	-0.137	-0.068	0.022	-0.022	-0.097	-0.012	-0.034
	upper	0.006	0.014	0.031	0.106	0.103	0.001	0.037	0.019
R. centralis	empirical	-0.140	-0.026	-0.008	**0.092**	0.032	-0.043	**0.028**	0.011
	lower	-0.230	-0.118	-0.057	0.051	-0.024	-0.100	0.003	-0.022
	upper	-0.061	0.052	0.039	0.143	0.087	0.009	0.061	0.047
R. dorbignyi	empirical	-0.124	-0.180	-0.152	**0.068**	**0.140**	-0.073	-0.034	-0.071
	lower	-0.201	-0.263	-0.189	0.029	0.107	-0.118	-0.053	-0.095
	upper	-0.048	-0.094	-0.112	0.108	0.177	-0.025	-0.015	-0.047
R. fernandezae	empirical	-0.127	-0.215	-0.073	**0.072**	**0.111**	-0.095	-0.009	-0.044
	lower	-0.186	-0.278	-0.109	0.043	0.074	-0.131	-0.027	-0.067
	upper	-0.072	-0.153	-0.037	0.100	0.148	-0.059	0.011	-0.020
R. granulosa	empirical	-0.046	-0.023	-0.003	**0.046**	**0.026**	-0.009	**0.014**	0.008
	lower	-0.075	-0.052	-0.021	0.030	0.007	-0.028	0.005	-0.003
	upper	-0.020	0.003	0.014	0.064	0.048	0.008	0.024	0.019
R. humboldti	empirical	-0.018	-0.033	0.016	**0.126**	-0.014	-0.053	**0.068**	**0.055**
	lower	-0.095	-0.110	-0.031	0.079	-0.084	-0.109	0.037	0.024
	upper	0.050	0.043	0.064	0.181	0.052	0.003	0.103	0.088
R. major	empirical	-0.093	-0.020	-0.030	**0.058**	**0.057**	-0.013	0.008	-0.003
	lower	-0.132	-0.059	-0.053	0.038	0.033	-0.037	-0.005	-0.017
	upper	-0.059	0.013	-0.007	0.078	0.083	0.010	0.021	0.010
R. margaritifera	empirical	-0.056	-0.083	-0.170	0.028	**0.127**	0.042	-0.026	-0.029
	lower	-0.200	-0.225	-0.245	-0.058	0.012	-0.043	-0.074	-0.075
	upper	0.070	0.077	-0.083	0.109	0.246	0.140	0.025	0.018
R. merianae	empirical	-0.070	-0.150	-0.048	**0.097**	**0.068**	-0.029	0.009	-0.007
	lower	-0.157	-0.236	-0.099	0.041	0.002	-0.088	-0.020	-0.042
	upper	0.017	-0.067	0.005	0.148	0.130	0.032	0.044	0.031
R. mirandaribeiroi	empirical	-0.058	0.000	0.023	**0.053**	**0.066**	0.005	**0.029**	0.020
	lower	-0.113	-0.058	-0.010	0.020	0.027	-0.029	0.009	-0.002
	upper	-0.005	0.055	0.057	0.087	0.107	0.042	0.049	0.040
R. pygmaea	empirical	-0.077	-0.089	-0.055	0.041	**0.154**	0.040	0.003	-0.002
	lower	-0.180	-0.188	-0.119	-0.027	0.087	-0.022	-0.033	-0.041
	upper	0.029	0.014	0.010	0.104	0.224	0.107	0.042	0.040

Table S9. Empirical and lower and upper bound IC95 values for AVG diff for the developmental units tested without allometric size variation. IC95 were constructed with 1,000 resampled P-matrices for each species. Significant AVG diff values are in bold (IC95 does not have zero).

DEVELOPMENTAL MODEL		AVG diff = [AVG +] - [AVG -]							
**no allometry**		**Branchial**	**Hyoid I**	**Hyoid II**	**Hyoid III**	**Mandibular I**	**Mandibular II**	**Mandibular III**	**Total II**
R. bergi	empirical	**0.385**	**0.068**	**0.101**	**0.113**	-0.014	0.001	0.009	**0.063**
	lower	0.202	0.014	0.025	0.036	-0.047	-0.041	-0.041	0.004
	upper	0.568	0.134	0.189	0.197	0.025	0.056	0.064	0.130
R. centralis	empirical	0.050	-0.018	0.042	0.029	-0.069	-0.078	-0.009	-0.046
	lower	-0.145	-0.057	-0.021	-0.025	-0.099	-0.108	-0.055	-0.098
	upper	0.265	0.025	0.114	0.095	-0.040	-0.045	0.043	0.010
R. dorbignyi	empirical	**0.281**	0.005	0.022	0.008	-0.004	-0.029	0.011	-0.013
	lower	0.158	-0.019	-0.011	-0.024	-0.024	-0.046	-0.021	-0.037
	upper	0.414	0.029	0.058	0.047	0.023	-0.007	0.044	0.013
R. fernandezae	empirical	**0.183**	**0.152**	**0.176**	**0.180**	-0.033	-0.031	-0.008	**0.079**
	lower	0.075	0.104	0.119	0.121	-0.056	-0.056	-0.040	0.046
	upper	0.296	0.200	0.237	0.237	-0.007	-0.005	0.026	0.114
R. granulosa	empirical	**0.171**	**0.070**	**0.135**	**0.115**	-0.015	-0.020	**0.042**	**0.062**
	lower	0.084	0.042	0.094	0.077	-0.032	-0.036	0.014	0.033
	upper	0.262	0.101	0.178	0.152	0.001	-0.003	0.069	0.089
R. humboldti	empirical	**0.195**	**0.042**	**0.066**	**0.068**	-0.002	-0.007	0.021	0.031
	lower	0.034	0.004	0.016	0.015	-0.031	-0.036	-0.021	-0.008
	upper	0.347	0.084	0.122	0.132	0.030	0.027	0.065	0.074
R. major	empirical	**0.164**	**0.029**	**0.069**	**0.063**	-0.027	-0.033	0.020	0.015
	lower	0.078	0.008	0.038	0.035	-0.041	-0.048	-0.005	-0.008
	upper	0.251	0.054	0.102	0.093	-0.012	-0.016	0.046	0.038
R. margaritifera	empirical	**0.218**	0.044	0.069	0.056	-0.070	-0.063	-0.027	-0.004
	lower	0.010	-0.010	-0.008	-0.017	-0.099	-0.106	-0.090	-0.059
	upper	0.431	0.102	0.150	0.132	-0.033	0.000	0.057	0.061
R. merianae	empirical	**0.347**	**0.067**	**0.163**	**0.073**	-0.014	-0.030	**0.036**	**0.078**
	lower	0.185	0.019	0.082	0.018	-0.043	-0.055	-0.011	0.029
	upper	0.486	0.114	0.240	0.125	0.019	-0.002	0.088	0.126
R. mirandaribeiroi	empirical	**0.253**	**0.056**	**0.104**	**0.106**	0.003	0.011	0.018	**0.074**
	lower	0.140	0.027	0.062	0.064	-0.019	-0.014	-0.012	0.045
	upper	0.364	0.089	0.151	0.152	0.027	0.040	0.051	0.108
R. pygmaea	empirical	0.167	0.044	**0.099**	**0.133**	-0.010	-0.009	0.052	0.050
	lower	-0.005	-0.001	0.032	0.060	-0.046	-0.049	-0.005	-0.005
	upper	0.328	0.093	0.174	0.205	0.029	0.036	0.114	0.102

Table S10. Empirical and IC95 values for AVG diff for the hormonal units tested without allometric size variation. IC95 were constructed with 1,000 resampled P-matrices for each species. Significant AVG diff values are in bold (IC95 does not have zero).

**HORMONAL MODEL**		**AVG diff = [AVG +] - [AVG -]**			
**no allometry**		**T3 +++**	**T3 ++**	**T3 +**	**Total**
R. bergi	empirical	**0.176**	**0.058**	0.060	**0.093**
	lower	0.048	0.006	-0.064	0.037
	upper	0.324	0.115	0.196	0.154
R. centralis	empirical	0.004	**0.107**	0.077	**0.103**
	lower	-0.100	0.032	-0.061	0.028
	upper	0.125	0.189	0.233	0.181
R. dorbignyi	empirical	**0.278**	**0.092**	0.029	**0.140**
	lower	0.180	0.047	-0.049	0.091
	upper	0.380	0.140	0.116	0.190
R. fernandezae	empirical	**0.154**	**0.043**	-0.034	**0.064**
	lower	0.078	0.014	-0.104	0.035
	upper	0.230	0.071	0.046	0.099
R. granulosa	empirical	**0.084**	**0.082**	0.031	**0.092**
	lower	0.027	0.057	-0.024	0.062
	upper	0.144	0.109	0.088	0.120
R. humboldti	empirical	**0.190**	**0.051**	-0.055	**0.076**
	lower	0.080	0.010	-0.121	0.030
	upper	0.303	0.098	0.021	0.125
R. major	empirical	**0.114**	**0.072**	**0.086**	**0.096**
	lower	0.053	0.045	0.025	0.066
	upper	0.170	0.100	0.152	0.126
R. margaritifera	empirical	0.061	0.047	-0.006	0.052
	lower	-0.127	-0.013	-0.057	-0.007
	upper	0.139	0.109	0.191	0.124
R. merianae	empirical	**0.164**	**0.108**	-0.036	**0.122**
	lower	0.059	0.053	-0.116	0.063
	upper	0.278	0.170	0.058	0.181
R. mirandaribeiroi	empirical	**0.095**	**0.087**	0.025	**0.098**
	lower	0.023	0.054	-0.037	0.062
	upper	0.166	0.125	0.097	0.137
R. pygmaea	empirical	**0.126**	**0.096**	0.109	**0.122**
	lower	0.019	0.037	-0.012	0.065
	upper	0.231	0.160	0.230	0.184

Table S11. Empirical and IC95 values for AVG diff for the functional units tested without allometric size variation. IC95 were constructed with 1,000 resampled P-matrices for each species. Significant AVG diff values are in bold (IC95 does not have zero).

**FUNCTIONAL MODEL**		**AVG diff = [AVG +] - [AVG -]**							
**no allometry**		**Neurocranium**	**Orbit**	**Roof**	**Snout**	**Suspensorium I**	**Suspensorium II**	**Total I**	**Total II**
R. bergi	empirical	**0.176**	**0.123**	**0.101**	**0.081**	0.018	-0.022	**0.097**	**0.077**
	lower	0.042	0.011	0.027	0.019	-0.072	-0.073	0.031	0.018
	upper	0.312	0.242	0.187	0.152	0.120	0.034	0.159	0.136
R. centralis	empirical	0.004	**0.208**	0.042	**0.182**	0.010	0.001	**0.132**	**0.120**
	lower	-0.093	0.047	-0.017	0.085	-0.075	-0.060	0.058	0.048
	upper	0.116	0.373	0.112	0.283	0.122	0.078	0.202	0.190
R. dorbignyi	empirical	**0.278**	**0.126**	-0.003	**0.099**	0.024	0.020	**0.093**	**0.084**
	lower	0.178	0.044	-0.031	0.054	-0.034	-0.020	0.059	0.053
	upper	0.370	0.223	0.032	0.149	0.089	0.069	0.127	0.115
R. fernandezae	empirical	**0.154**	0.016	**0.134**	**0.157**	**0.099**	0.004	**0.159**	**0.140**
	lower	0.083	-0.047	0.083	0.109	0.029	-0.037	0.117	0.101
	upper	0.238	0.087	0.186	0.208	0.171	0.046	0.199	0.177
R. granulosa	empirical	**0.084**	**0.108**	**0.069**	**0.127**	**0.079**	**0.048**	**0.108**	**0.101**
	lower	0.028	0.052	0.037	0.093	0.027	0.012	0.075	0.070
	upper	0.142	0.170	0.101	0.164	0.133	0.086	0.139	0.132
R. humboldti	empirical	**0.190**	**0.200**	**0.067**	**0.130**	**0.155**	**0.092**	**0.167**	**0.164**
	lower	0.083	0.085	0.019	0.064	0.060	0.029	0.112	0.111
	upper	0.302	0.313	0.124	0.197	0.254	0.160	0.222	0.216
R. major	empirical	**0.114**	**0.130**	**0.067**	**0.094**	0.023	0.025	**0.087**	**0.083**
	lower	0.058	0.065	0.036	0.065	-0.018	-0.004	0.060	0.058
	upper	0.175	0.199	0.097	0.128	0.073	0.057	0.114	0.108
R. margaritifera	empirical	0.061	0.063	0.037	**0.082**	-0.008	-0.012	**0.077**	**0.070**
	lower	-0.054	-0.064	-0.029	0.007	-0.102	-0.092	0.016	0.010
	upper	0.202	0.211	0.108	0.157	0.121	0.085	0.135	0.129
R. merianae	empirical	**0.164**	0.012	0.034	**0.141**	0.004	0.040	**0.084**	**0.087**
	lower	0.057	-0.075	-0.012	0.075	-0.064	-0.016	0.031	0.035
	upper	0.277	0.102	0.085	0.213	0.078	0.107	0.136	0.141
R. mirandaribeiroi	empirical	**0.095**	**0.143**	**0.087**	**0.110**	0.056	0.025	**0.112**	**0.100**
	lower	0.027	0.064	0.041	0.068	-0.006	-0.012	0.075	0.066
	upper	0.163	0.223	0.132	0.160	0.118	0.066	0.151	0.136
R. pygmaea	empirical	**0.126**	0.093	**0.087**	**0.119**	0.046	0.041	**0.116**	**0.120**
	lower	0.020	-0.015	0.022	0.055	-0.037	-0.017	0.064	0.069
	upper	0.239	0.207	0.157	0.186	0.137	0.111	0.172	0.172

Table S12. Empirical and lower and upper bound IC95 values for AVG diff for the developmental units tested without isometric size variation. IC95 were constructed with 1,000 resampled P-matrices for each species. Significant AVG diff values are in bold (IC95 does not have zero).

DEVELOPMENTAL MODEL		AVG diff = [AVG +] - [AVG -]							
no isometry		Branchial	Hyoid I	Hyoid II	Hyoid III	Mandibular I	Mandibular II	Mandibular III	Total II
R. bergi	empirical	**0.356**	0.016	0.056	0.034	**0.058**	**0.076**	**0.079**	**0.089**
	lower	0.161	-0.016	-0.002	-0.015	0.018	0.021	0.019	0.030
	upper	0.537	0.059	0.127	0.095	0.107	0.140	0.146	0.154
R. centralis	empirical	0.107	-0.006	0.042	0.011	-0.026	-0.042	0.015	-0.021
	lower	-0.095	-0.036	-0.013	-0.040	-0.049	-0.065	-0.029	-0.067
	upper	0.317	0.034	0.108	0.070	0.001	-0.013	0.063	0.029
R. dorbignyi	empirical	**0.377**	-0.012	0.008	-0.013	0.021	**0.045**	0.015	**0.036**
	lower	0.237	-0.028	-0.019	-0.040	-0.001	0.014	-0.013	0.007
	upper	0.507	0.008	0.042	0.017	0.046	0.080	0.044	0.069
R. fernandezae	empirical	**0.254**	**0.069**	**0.083**	**0.087**	**0.071**	**0.125**	**0.096**	**0.141**
	lower	0.139	0.038	0.047	0.045	0.042	0.086	0.061	0.105
	upper	0.371	0.102	0.124	0.130	0.103	0.165	0.131	0.176
R. granulosa	empirical	**0.209**	**0.031**	**0.093**	**0.056**	**0.044**	**0.039**	**0.091**	**0.081**
	lower	0.115	0.012	0.059	0.025	0.024	0.015	0.058	0.053
	upper	0.305	0.053	0.129	0.088	0.065	0.063	0.124	0.110
R. humboldti	empirical	**0.209**	0.010	**0.062**	0.030	**0.049**	**0.047**	**0.061**	**0.070**
	lower	0.065	-0.013	0.020	-0.009	0.018	0.012	0.014	0.030
	upper	0.356	0.041	0.116	0.078	0.086	0.087	0.113	0.114
R. major	empirical	**0.222**	0.009	**0.046**	**0.026**	**0.027**	**0.036**	**0.052**	**0.054**
	lower	0.134	-0.007	0.021	0.003	0.010	0.015	0.026	0.029
	upper	0.310	0.026	0.072	0.051	0.044	0.059	0.077	0.077
R. margaritifera	empirical	0.085	-0.030	0.010	-0.010	**0.053**	**0.093**	**0.101**	**0.069**
	lower	-0.110	-0.059	-0.039	-0.056	0.010	0.031	0.030	0.013
	upper	0.298	0.002	0.072	0.039	0.104	0.166	0.182	0.130
R. merianae	empirical	**0.294**	0.029	**0.120**	0.030	0.022	0.014	**0.082**	**0.082**
	lower	0.138	-0.001	0.059	-0.008	-0.004	-0.014	0.034	0.036
	upper	0.444	0.063	0.186	0.075	0.052	0.049	0.138	0.131
R. mirandaribeiroi	empirical	**0.253**	0.007	**0.039**	**0.036**	**0.064**	**0.062**	**0.075**	**0.071**
	lower	0.142	-0.011	0.010	0.004	0.035	0.033	0.041	0.042
	upper	0.364	0.029	0.070	0.070	0.092	0.094	0.112	0.101
R. pygmaea	empirical	**0.279**	0.012	**0.059**	**0.068**	**0.058**	**0.080**	**0.096**	**0.094**
	lower	0.111	-0.016	0.011	0.010	0.020	0.027	0.041	0.043
	upper	0.453	0.046	0.110	0.129	0.099	0.130	0.152	0.138

Table S13. Empirical and IC95 values for AVG diff for the hormonal units tested without isometric size variation. IC95 were constructed with 1,000 resampled P-matrices for each species. Significant AVG diff values are in bold (IC95 does not have zero).

**HORMONAL MODEL**		**AVG diff = [AVG +] - [AVG -]**			
**no isometry**		**T3 +++**	**T3 ++**	**T3 +**	**Total**
R. bergi	empirical	**0.226**	**0.069**	0.043	**0.111**
	lower	0.082	0.021	-0.073	0.049
	upper	0.366	0.121	0.181	0.172
R. centralis	empirical	0.048	**0.067**	0.047	**0.073**
	lower	-0.050	0.006	-0.081	0.008
	upper	0.169	0.129	0.191	0.146
R. dorbignyi	empirical	**0.292**	**0.068**	0.073	**0.127**
	lower	0.187	0.036	-0.009	0.078
	upper	0.392	0.110	0.156	0.176
R. fernandezae	empirical	**0.212**	0.009	**0.083**	**0.061**
	lower	0.129	-0.009	0.009	0.032
	upper	0.298	0.030	0.163	0.093
R. granulosa	empirical	**0.139**	**0.039**	**0.062**	**0.070**
	lower	0.085	0.020	0.005	0.044
	upper	0.198	0.059	0.122	0.096
R. humboldti	empirical	**0.212**	**0.073**	-0.038	**0.101**
	lower	0.108	0.029	-0.104	0.048
	upper	0.320	0.117	0.037	0.155
R. major	empirical	**0.161**	**0.053**	**0.113**	**0.092**
	lower	0.103	0.031	0.049	0.065
	upper	0.221	0.075	0.177	0.121
R. margaritifera	empirical	-0.003	**0.057**	**0.205**	**0.074**
	lower	-0.092	0.001	0.046	0.014
	upper	0.103	0.119	0.366	0.142
R. merianae	empirical	**0.159**	**0.082**	0.035	**0.107**
	lower	0.058	0.041	-0.049	0.057
	upper	0.273	0.125	0.140	0.165
R. mirandaribeiroi	empirical	**0.171**	**0.046**	**0.097**	**0.086**
	lower	0.099	0.020	0.023	0.052
	upper	0.257	0.072	0.168	0.122
R. pygmaea	empirical	**0.172**	0.035	**0.174**	**0.087**
	lower	0.051	0.000	0.052	0.032
	upper	0.296	0.076	0.295	0.138

Table S14. Empirical and lower and upper bound IC95 values for AVG diff for the functional units tested without isometric size variation. IC95 were constructed with 1,000 resampled P-matrices for each species. Significant AVG diff values are in bold (IC95 does not have zero).

**FUNCTIONAL MODEL**		**AVG diff = [AVG +] - [AVG -]**							
**no isometry**		**Neurocranium**	**Orbit**	**Roof**	**Snout**	**Suspensorium I**	**Suspensorium II**	**Total I**	**Total II**
R. bergi	empirical	**0.226**	**0.195**	0.021	**0.069**	0.066	0.013	**0.085**	**0.064**
	lower	0.094	0.063	-0.029	0.017	-0.036	-0.039	0.034	0.015
	upper	0.378	0.329	0.079	0.127	0.192	0.077	0.141	0.120
R. centralis	empirical	0.048	**0.190**	0.024	**0.096**	0.018	0.017	**0.078**	**0.068**
	lower	-0.050	0.030	-0.026	0.028	-0.069	-0.049	0.021	0.008
	upper	0.156	0.362	0.088	0.175	0.120	0.088	0.138	0.127
R. dorbignyi	empirical	**0.292**	**0.155**	-0.009	**0.069**	**0.090**	-0.006	**0.087**	**0.054**
	lower	0.192	0.073	-0.037	0.030	0.024	-0.037	0.053	0.021
	upper	0.391	0.250	0.023	0.109	0.165	0.034	0.124	0.088
R. fernandezae	empirical	**0.212**	0.007	**0.048**	**0.082**	**0.282**	**0.071**	**0.130**	**0.100**
	lower	0.137	-0.051	0.013	0.047	0.196	0.031	0.094	0.066
	upper	0.296	0.073	0.086	0.118	0.367	0.113	0.166	0.133
R. granulosa	empirical	**0.139**	**0.126**	0.022	**0.091**	**0.088**	**0.075**	**0.084**	**0.081**
	lower	0.082	0.064	-0.003	0.061	0.040	0.040	0.058	0.057
	upper	0.198	0.184	0.046	0.122	0.144	0.114	0.110	0.109
R. humboldti	empirical	**0.212**	**0.159**	0.029	**0.168**	**0.122**	**0.095**	**0.165**	**0.164**
	lower	0.108	0.052	-0.013	0.100	0.033	0.035	0.109	0.107
	upper	0.318	0.272	0.078	0.237	0.214	0.160	0.220	0.220
R. major	empirical	**0.161**	**0.132**	**0.034**	**0.076**	**0.051**	0.025	**0.074**	**0.062**
	lower	0.105	0.069	0.008	0.049	0.007	-0.003	0.049	0.038
	upper	0.220	0.194	0.062	0.105	0.098	0.054	0.100	0.086
R. margaritifera	empirical	-0.003	0.022	-0.050	0.036	**0.172**	**0.084**	0.037	0.037
	lower	-0.094	-0.095	-0.089	-0.017	0.040	0.005	-0.008	-0.010
	upper	0.106	0.142	-0.010	0.096	0.304	0.168	0.086	0.087
R. merianae	empirical	**0.159**	0.032	0.008	**0.118**	0.050	0.051	**0.073**	**0.070**
	lower	0.063	-0.048	-0.026	0.062	-0.023	-0.003	0.026	0.026
	upper	0.256	0.137	0.044	0.172	0.130	0.104	0.118	0.117
R. mirandaribeiroi	empirical	**0.171**	**0.170**	**0.032**	**0.072**	**0.103**	**0.048**	**0.092**	**0.077**
	lower	0.094	0.093	0.000	0.041	0.042	0.011	0.058	0.044
	upper	0.251	0.251	0.066	0.108	0.171	0.095	0.127	0.112
R. pygmaea	empirical	**0.172**	**0.119**	0.047	**0.053**	**0.115**	**0.062**	**0.085**	**0.081**
	lower	0.065	0.014	-0.004	0.011	0.021	0.004	0.040	0.038
	upper	0.295	0.237	0.107	0.100	0.216	0.127	0.132	0.123

Table S15. Dissimilarity matrices among species P-matrices with size variation (below diagonal) and without isometric size variation (above diagonal). Species P-matrices were compared using Random Skewers (RS), which is interpreted as the similarity (S) in the species average response to selection. The similarity indexes were transformed into dissimilarity indexes by taking the square root of (1 – S).

	1	2	3	4	5	6	7	8	9	10
**1. *R. bergi***		0.52	0.42	0.49	0.43	0.48	0.40	0.44	0.45	0.49
**2. *R. centralis***	0.22		0.52	0.55	0.47	0.50	0.45	0.52	0.52	0.54
**3. *R. dorbignyi***	0.32	0.30		0.43	0.42	0.49	0.39	0.43	0.41	0.49
**4. *R. fernandezae***	0.35	0.35	0.21		0.43	0.45	0.42	0.46	0.45	0.51
**5. *R. granulosa***	0.24	0.28	0.32	0.33		0.33	0.28	0.33	0.31	0.39
**6. *R. humboldti***	0.27	0.27	0.35	0.34	0.24		0.37	0.40	0.37	0.45
**7. *R. major***	0.23	0.22	0.25	0.27	0.17	0.22		0.35	0.31	0.42
**8. *R. merianae***	0.30	0.30	0.30	0.33	0.29	0.29	0.26		0.38	0.47
**9. *R. mirandaribeiroi***	0.26	0.28	0.29	0.28	0.20	0.22	0.18	0.21		0.44
**10. *R. pygmaea***	0.32	0.32	0.26	0.29	0.31	0.32	0.24	0.28	0.26	

Table S16. Dissimilarity matrices among P-matrices using Relative Eigenanalysis. Distances between pairs of species P-matrices were calculated as the square root of the sum of their squared log-transformed relative eigenvalues, after multiplying a species matrix A by the inverse of a species matrix B (Booksetein & Mittereocker 2009). Values below the diagonal are for P-matrices with size variation and values above the diagonal are for P-matrices without isometric size variation.

	1	2	3	4	5	6	7	8	9	10
**1. *R. bergi***		3.11	1.80	2.32	1.87	1.91	1.55	1.78	1.81	1.99
**2. *R. centralis***	2.23		2.98	4.19	2.34	2.53	2.56	2.71	2.75	2.92
**3. *R. dorbignyi***	2.40	2.43		2.35	1.81	1.95	1.58	1.75	1.69	2.08
**4. *R. fernandezae***	4.21	4.16	3.16		2.69	2.64	2.41	2.58	2.48	2.67
**5. *R. granulosa***	2.22	2.16	2.24	3.21		1.05	1.09	1.26	1.24	1.61
**6. *R. humboldti***	2.90	2.79	2.53	2.46	1.97		1.24	1.38	1.35	1.72
**7. *R. major***	2.00	1.97	1.80	3.15	1.26	1.92		1.34	1.10	1.59
**8. *R. merianae***	2.32	2.34	1.84	3.23	1.85	2.00	1.66		1.43	1.83
**9. *R. mirandaribeiroi***	2.79	2.71	2.19	2.41	1.77	1.41	1.64	1.66		1.79
**10. *R. pygmaea***	2.29	2.42	1.79	3.36	1.95	2.19	1.67	1.67	1.98	

Table S17. Results of variation partitioning models when using Relative Eigenanalysis (RE) to compare species P-matrices. For species P-matrices with size variation, the model constructed has PCoA 2 of the dissimilarity matrix as the dependent variable (accounting for 47% of variation), which has significant correlations with both PCoA 1 of phylogeny (cor = 0.74 P = 0.013) and with climatic PC1 (cor = -0.7 P = 0.024). For species P-matrices without isometric size variation, the model had as dependent variable the PCoA 1 of the dissimilarity matrix and PCoA 1 (cor = 0.75 P = 0.013) phylogeny and climatic PC1 (cor = -0.72 P = 0.02) as independent factors.

**PCoA Relative Eigenanalysis Dissimilarity**			
with size	Adjusted R squared	F	P
Phylogeny	**0.5**	**10.17**	**0.014**
Climate	**0.43**	**7.7**	**0.027**
Phylogeny| Climate	0.01	1.16	0.32
Climate| Phylogeny	-0.07	0.048	0.85
Phylogeny: Climate	0.49	-	-
no isometric size	Adjusted R squared	F	P
Phylogeny	**0.5**	**10.11**	**0.003**
Climate	**0.45**	**8.4**	**0.007**
Phylogeny| Climate	-0.07	0.89	0.37
Climate| Phylogeny	-0.06	0.14	0.7
Phylogeny: Climate	0.51	-	-

Table S18. Contrast in climatic variables between the most basal and the most derived species in the toad phylogeny. The species having the higher contrasts in the values of the climatic variables relevant in the climatic PC1 are colored in gray. *R. cent = R. centralis; R. humb. = R. humboldti, R. meri. = R. merianae, R. gran. = R. granulosa, R. mira. = R. mirandaribeiroi, R. maj. = R. major, R. berg. = R. bergi, R. pygm. = R. pygmaea, R. dorb. = R. dorbignyi, R. fern = R. fernandezae*.

Variables	*R. cent.*	*R. humb.*	*R. meri.*	*R. gran.*	*R. mira.*	*R. maj.*	*R. berg.*	*R. pygm.*	*R. dorb.*	*R. fern.*
Temperature Seasonality (oC)	5.8	5.9	5.1	13.2	9.0	23.4	38.3	18.6	43.8	43.6
Minimum Temperature of Coldest Month (oC)	21.9	20.3	21.2	16.8	16.8	14.2	9.6	15.8	5.9	7.1
Mean Temperature of Driest Quarter (oC)	27.0	26.1	26.8	23.2	24.7	21.4	16.5	20.7	14.7	13.8
Mean Temperature of Coldest Quarter (oC)	26.2	25.3	25.8	22.1	24.1	20.9	16.4	20.7	11.0	12.5
Precipitation of Wettest Month (mm)	322.8	265.4	326.7	180.2	295.9	225.5	149.4	156.3	112.3	128.9
Precipitation Seasonality (mm)	66.8	56.5	58.6	72.0	78.7	61.5	46.9	43.4	16.7	23.5
Precipitation of Wettest Quarter (mm)	816.2	700.7	889.5	477.0	810.5	621.1	406.7	421.3	310.4	347.2

Table S19. Allometric coefficients for the bones composing the mandibular I, the suspensorium I and the T3 + units. We log-transformed all distances and regressed each of them against log (skull length) to calculate the regression slope, which corresponds to the allometric coefficients. Coefficients in bold indicate positive allometry. Columns coloured in gray are the distances composing the suspensorium I functional unit.

Bones	squamosal	maxilla	squamosal	maxilla	pterygoid	pterygoid	mandible	neopalatine
R. centralis	**1.1**	0.98	**1.2**	0.92	0.8	**1.5**	1.0	0.98
R. humboldti	**1.5**	**1.14**	**1.3**	**1.06**	**1.1**	**1.1**	1.0	**1.15**
R. merianae	**1.4**	0.85	**1.1**	0.96	0.9	**1.1**	0.9	1.01
R. granulosa	**1.2**	**1.09**	**1.1**	**1.10**	0.9	**1.2**	1.0	**1.13**
R. mirandaribeiroi	**1.3**	0.92	1.0	0.94	1.0	1.0	0.9	0.95
R. major	**1.2**	**1.10**	**1.1**	0.99	1.0	**1.1**	1.0	**1.08**
R. bergi	**1.1**	1.04	**1.4**	**1.17**	0.8	**1.2**	0.9	**1.21**
R. pygmaea	1.0	**1.07**	1.0	0.87	**1.1**	0.9	1.0	0.78
R. dorbignyi	**1.4**	**1.06**	1.0	1.02	1.0	**1.3**	**1.1**	1.03
R. fernandezae	**1.2**	**1.13**	1.0	**1.05**	1.0	**1.2**	**1.1**	**1.07**

Table S20. Mean estimated correlations for preferred developmental and functional modularity models without isometric size variation. Notice that the more integrated functional module is the neurocranium** followed by the orbit for the species that the functional model was the best supported. For the species in which one of the developmental model was the preferred one, the branchial or mandibular III** modules are the more integrated.

**No isometric size**		**rho**				
**Species**	**Model**	**neurocranium****	**snout**	**orbit**	**suspensorium II**	**between**
R. centralis	Functional VII	0.26	0.03	0.16	-0.01	-0.07
R. humboldti	Functional VII	0.50	0.11	0.11	0.01	-0.09
R. granulosa	Functional VII	0.44	0.02	0.08	-0.03	-0.09
R. mirandaribeiroi	Functional VII	0.20	0.03	0.12	0.04	-0.07
R. major	Functional VII	0.41	0.02	0.09	-0.05	-0.07
R. bergi	Functional VII	0.50	0.03	0.17	-0.04	-0.07
	**Model**	**branchial**	**hyoid II**	**mandibular III****	**between**	
R. merianae	Developmental IV	0.25	0.07	0.04	-0.10	
R. pygmaea	Developmental V	0.23	0.13	0.04	-0.11	
R. fernandezae	Developmental IV	0.21	0.04	0.19	-0.13	
R. margaritifera	Developmental IV	0.06	-0.01	0.13	-0.08	
	**Model**	**T3 +**	**T3 ++**	**T3 +++**	**between**	
R. dorbignyi	Hormonal III	0.03	0.01	0.29	-0.09	

Table S21. Allometric coefficients for the occipital and parasphenoid bones. We log-transformed all distances and regressed each of them against log (skull length) to calculate the regression slope, which corresponds to the allometric coefficients. Coefficients in bold indicate positive allometry. Columns colored in gray are from the species that have positive coefficients in climatic PC1, whereas columns in white are from species with negative coefficients in climatic PC1 (see Fig. 5B, C).

Allometric coefficients	occipital	parasphenoid	parasphenoid
R. centralis	0.99	**1.25**	**1.28**
R. humboldti	**1.09**	**1.14**	**1.08**
R. merianae	**1.28**	0.99	0.91
R. granulosa	**1.14**	1.01	1.01
R. mirandaribeiroi	0.91	0.88	0.78
R. major	**1.08**	0.96	0.93
R. bergi	0.85	1.04	1.03
R. pygmaea	0.77	0.77	0.64
R. dorbignyi	0.84	1.02	0.99
R. fernandezae	0.94	0.98	0.96

Supplementary Figures

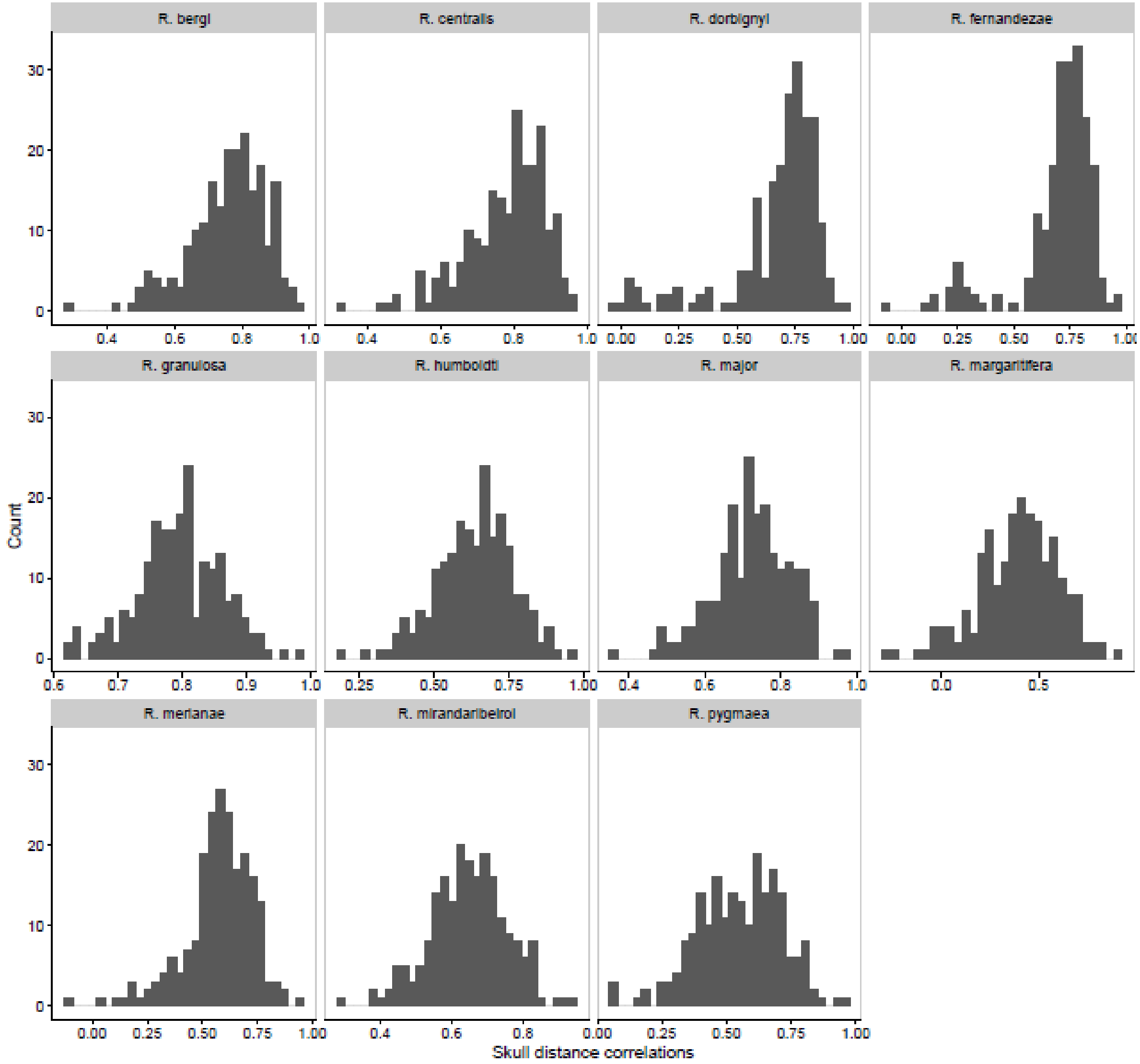

Figure S1. Distribution of skull distance correlations in all the toad species. Twenty-one linear distances corresponding to dimensions of single bones were calculated from 3D landmarks placed in the skulls of the species. The correlation values were extracted from species Pearson product-moment correlation phenotypic matrices which had variation in mean skull distances due to sexual dimorphism and geographic localities removed.

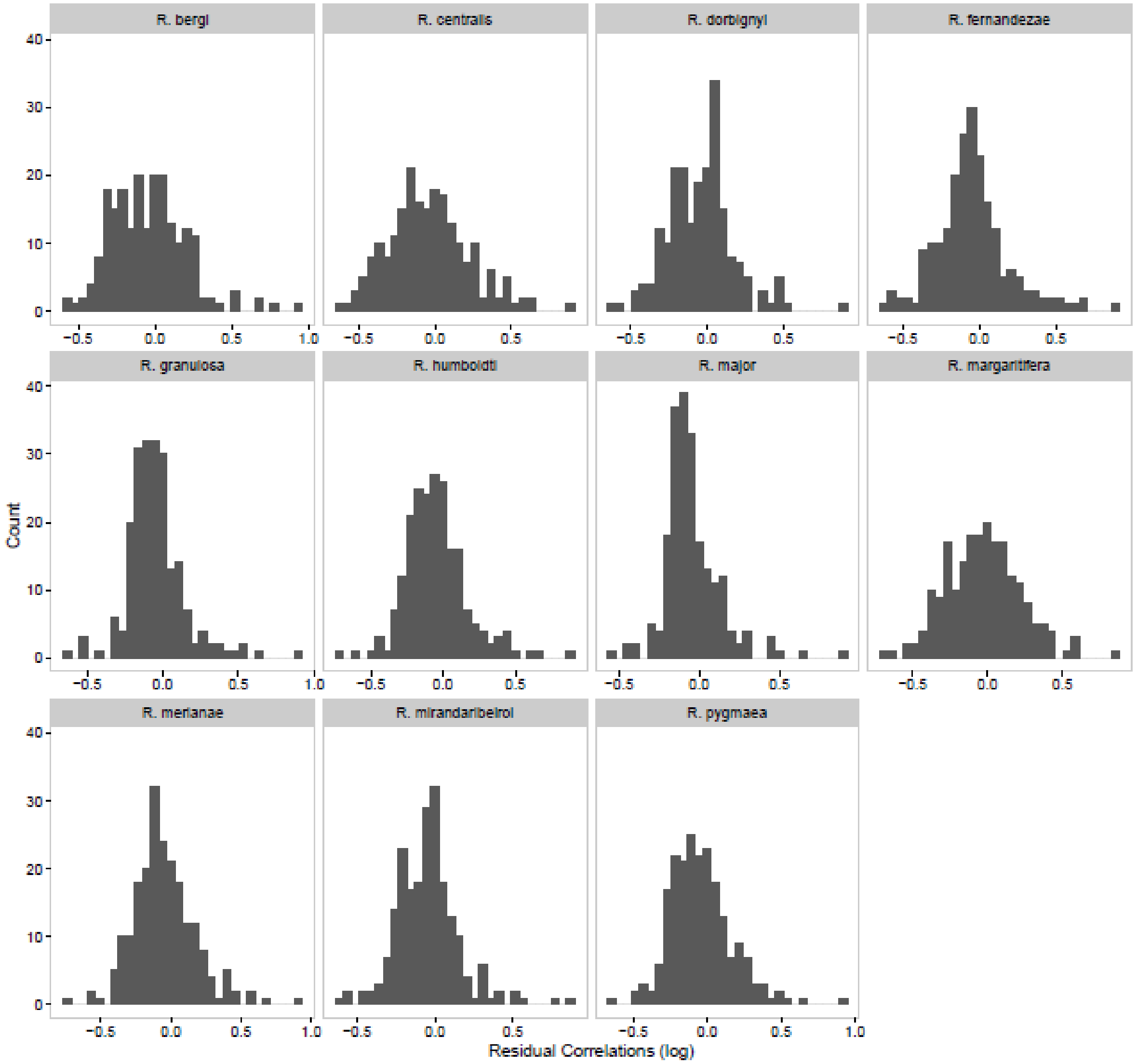

Figure S2. Distribution of skull distance correlations after removal of isometric size variation. After removing isometric size variation from species phenotypic matrices, all species have similar distributions, averaging on zero and ranging from -0.5 to 0.5. The correlation distributions when allometric size was removed are very similar to the ones showed.

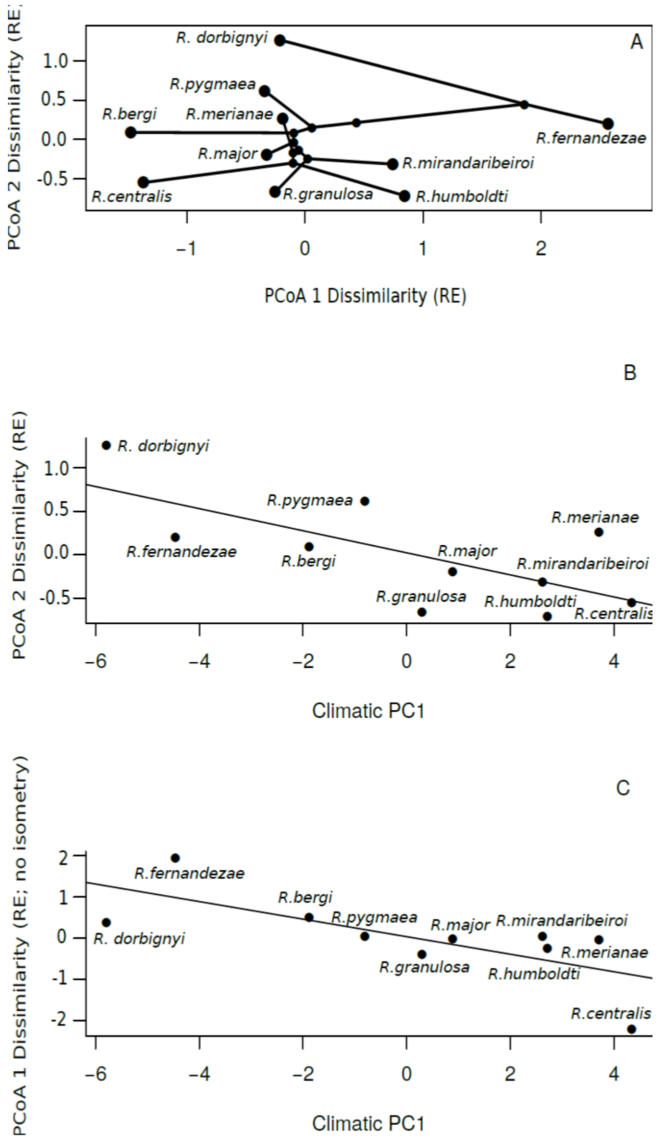

Figure S3. Principal coordinate axis (PCoA) of P-matrix dissimilarity analyzed with Relative Eigenanalysis (A) and associations between dissimilarity PCo axes and climatic variation among species with size variation (B) and without isometric size variation (C). The dissimilarity matrix was constructed by using Bookstein & Mitteroecker's (2009) distance. For both dissimilarity PCo axes, with or without isometric size variation, climatic variation structured by phylogheny explains part of the P-matrix variation.
